# Impact of collegial midwifery assistance during second stage of labour on women’s experience: a follow-up from the Swedish Oneplus randomised controlled trial

**DOI:** 10.1136/bmjopen-2023-077458

**Published:** 2024-07-26

**Authors:** Cecilia Häggsgård, Malin Edqvist, Pia Teleman, Helena Tern, Christine Rubertsson

**Affiliations:** 1 Department of Obstetrics and Gynecology, Skane University Hospital, Lund, Sweden; 2 Department of Health Sciences, Faculty of Medicine, Lund University, Lund, Sweden; 3 Clinical Epidemiology Unit, Department of Medicine Solna, Karolinska Institutet, Stockholm, Sweden; 4 Department of Women’s Health and Health Professions, Karolinska University Hospital, Stockholm, Sweden; 5 Department of Clinical Sciences, Lund University, Lund, Sweden; 6 Department of Obstetrics and Gynecology, Skane University Hospital, Malmö, Sweden

**Keywords:** obstetrics, randomized controlled trial, patient reported outcome measures

## Abstract

**Objective:**

To compare experiences of the second stage of labour in women randomised to assistance by one or by two midwives to reduce severe perineal trauma (SPT).

**Design:**

Analysis of a secondary outcome within the Swedish Oneplus multicentre randomised trial.

**Setting:**

Five obstetric units in Sweden between December 2018 and March 2020.

**Participants:**

Inclusion criteria in the Oneplus trial were women opting for their first vaginal birth from gestational week 37+0 with a singleton pregnancy and a live fetus in the vertex presentation. Further inclusion criteria were language proficiency in Swedish, English, Arabic or Farsi. Exclusion criteria were multiple pregnancies, intrauterine fetal demise and planned caesarean section. Of the 3059 women who had a spontaneous vaginal birth, 2831 women had consented to participate in the follow-up questionnaire.

**Interventions:**

Women were randomly assigned (1:1) to assistance by two midwives (intervention group) or one midwife (standard care) when reaching the second stage of labour.

**Outcome measures:**

Data were analysed by intention to treat. Comparisons between intervention and standard care regarding experiences of the second stage of labour were evaluated with items rated on Likert scales. The Student’s t-test was used to calculate mean differences with 95% CIs.

**Results:**

In total 2221 (78.5%) women responded to the questionnaire. There were no statistically significant differences regarding women’s experiences of being in control, feelings of vulnerability or pain. Women randomised to be assisted by two midwives agreed to a lesser extent that they could handle the situation during the second stage (mean 3.18 vs 3.26, 95% CI 0.01 to 0.15). Conducted subgroup analyses revealed that this result originated from one of the study sites.

**Conclusions:**

The intervention’s lack of impact on the experience of the second stage is of importance considering the reduction in SPT when being assisted by two midwives.

**Trial registration number:**

NCT03770962.

**STRENGTHS AND LIMITATIONS OF THIS STUDY**

- The randomised design is considered the most rigorous and robust design for determining cause and effect.
- The high response rate indicates the importance of the topic for women.
- It was not possible to blind the participants or midwives due to the nature of the intervention which may have influenced women’s responses.
- The items in the questionnaire were developed for the purpose of the study and have not undergone full validation, including psychometric properties.

## Introduction

 The second stage of labour has been described by women as an intensive phase that evokes a variety of emotions ranging from pain and fear to power and strength^1^ and women have expressed that establishing new relationships with caregivers, at this stage, is difficult.^1^,^3^ Caregivers are responsible for securing the well-being of the woman and the fetus, including prevention of perineal injuries. Culmination of the second stage of labour with the birth of the child can be described as a transformative state between pregnancy and motherhood requiring enhanced involvement and support from carers.^1 4^ For many women, fear of birth is connected with fear of sustaining perineal trauma^5^ which is further associated with negative birth experiences.^6^

Severe perineal trauma (SPT) is a major global issue.^7 8^ It involves trauma to the external and/or internal anal sphincter (third degree tear) and the anorectal mucosa (fourth degree tear).^9^ Risk factors for SPT include nulliparity, first vaginal birth after caesarean section, instrumental birth, increased fetal birth weight, increased maternal age and Asian ethnicity.^10 11^ In Sweden and other Scandinavian countries, the incidence of SPT increased during the early 2000s.^12^ This led to a major focus on prevention of SPT, as this injury is associated with pain, dyspareunia and postponed coital resumption^13 14^ and it is also the primary contributor to anal incontinence among women later in life.^13^ Among quality improvement initiatives to reduce SPT,^15^ a clinical practice termed collegial midwifery assistance, started to spread in the Scandinavian countries. This practice entails two midwives assisting the woman during the late second stage of labour with the aim to prevent SPT and has been implemented at many obstetric units in Sweden.

A clinical trial was performed between 2018 and 2020 to test the hypothesis that collegial midwifery assistance during the second stage of labour reduces SPT.^16^ Results showed that the presence of a second midwife during the late second stage reduced the risk of SPT by 30% for women who experienced spontaneous vaginal birth for the first time. In the trial, types of collegial midwifery assistance could be the mere presence in the birthing room of the second midwife or provision of active support including communication with the woman, interpretation of cardiotocography patterns, giving feedback on preventive methods used and assisting with manual perineal protection.^16^ The results also showed that the median time for collegial assistance was 15 min. The midwives were instructed to use the established prevention models^17 18^ at their respective labour wards, and for the second midwife to be ready to assist the primary midwife if asked and to support the birthing woman if needed.^16^ Since previous research shows that it can be difficult to establish new relationships during the second stage,^1^ it can be assumed that the assistance of a second midwife may influence the women’s experiences of the second stage negatively. Therefore, this was predefined as a secondary outcome in the trial.^19^ The objective of this study was to compare experiences of the second stage of labour between women assigned to collegial midwifery assistance or to standard care during the late second stage of labour in the Oneplus trial.

## Methods

### Study design and participants

**Figure 1:**
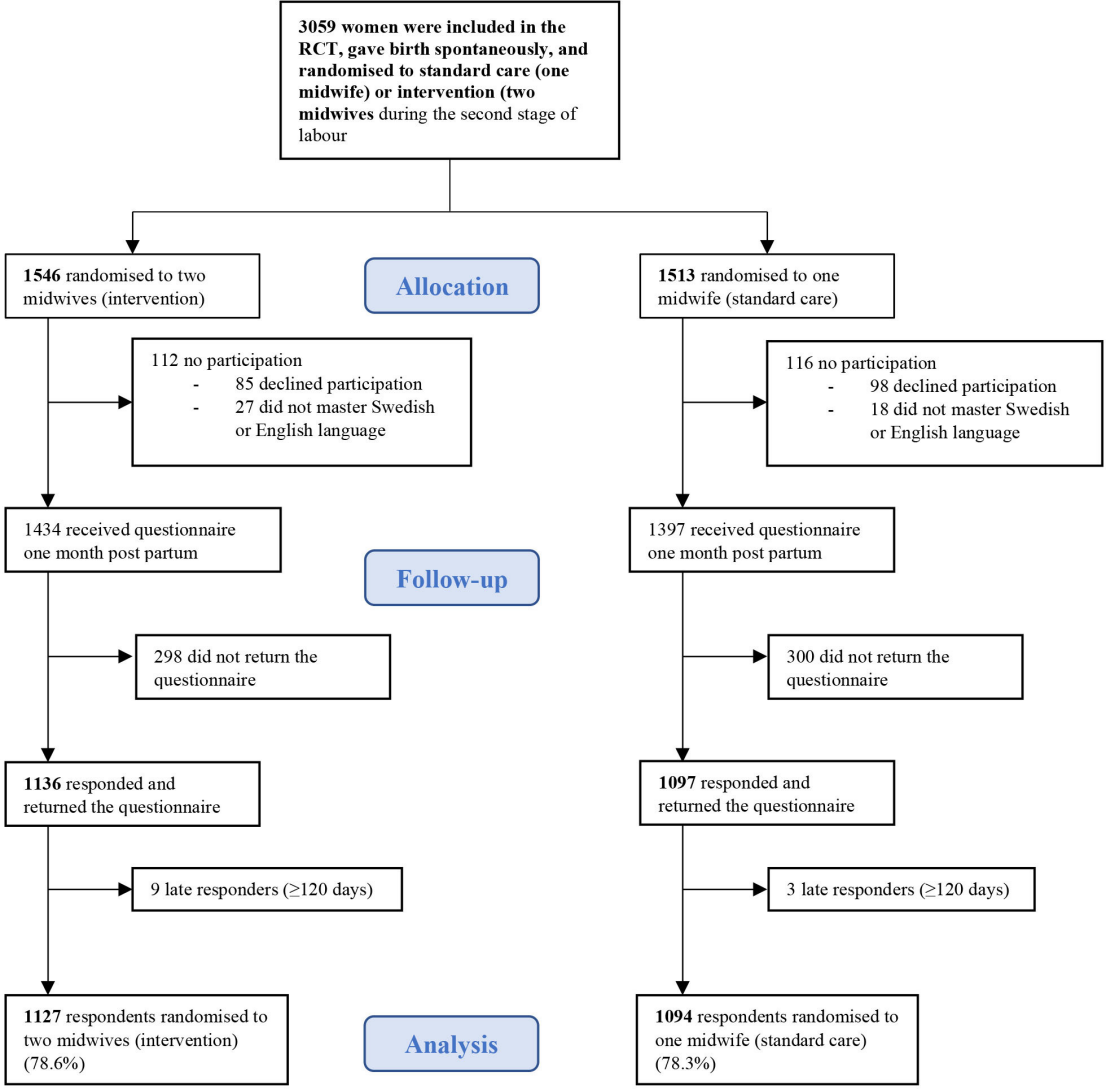
Flowchart of the women who were randomised in the Oneplus trial and asked about consent to the follow-up questionnaire. RCT, randomised controlled trial.

The Oneplus trial was an open-label multicentre randomised controlled trial conducted at five obstetric units in Sweden.^16^ Data collection for the primary endpoint of the trial took place between December 2018 and March 2020. Women were randomly assigned (1:1) to either the intervention group, that is, assistance by two midwives during the active second stage of labour or in the standard care group, assistance by one midwife. In total, 3059 women gave birth spontaneously: 1546 in the intervention group were assisted by two midwives and 1513 in the standard care group were assisted by one midwife. The study has been described in detail elsewhere.^16^ Inclusion criteria were women aged between 18 and 47 who opted for their first vaginal birth, from gestational week 37+0 with a singleton live fetus in the vertex presentation. Further inclusion criteria in the Oneplus trial were language proficiency in Swedish, English, Arabic or Farsi as study information was only available in these languages. As the questionnaire was only available in Swedish and English, women who did not master Swedish or English were not included in the current study. Of the total study population of 3059 women, 2831 (92%) provided consent to participate in a follow-up questionnaire 1 month after birth (figure 1).

### Intervention and standard care

In Sweden, midwives are the primary caregivers during pregnancy, birth and the postnatal period. In uncomplicated labour and birth, women are assisted by midwives and healthcare assistants. In the event of complications or deviations from the normal, midwives work in close collaboration with obstetricians who perform operative births.^20^ All study sites in the trial worked actively with different models for prevention of SPT.^17 18^ Midwives were instructed to work with the prevention models that they already used and that the purpose of the second midwife was to assist the primary midwife in preventing SPT. The primary midwife was told to ask for the second midwife to be present in the birthing room when the active phase of the second stage had started and when the presenting part was visible, and they should be ready to assist if asked.^16^

### Questionnaire development

A study specific questionnaire was developed to assess secondary outcomes in the trial; experiences of the second stage of labour and collegial midwifery assistance. Furthermore, questions regarding sociodemographic background and history of physical and mental health (online supplemental appendix). For the purpose of the present study, the items regarding experiences of the second stage were analysed (box 1).

Box 1 **The 4-point Likert scales range from 1 (strongly agree) to 4 (disagree). The responses were reversed in order for higher scores to indicate greater agreement with the statement**

**Items rated on a 4-pointLikertscale**

- I felt strong during the second stage of labour.
- I could handle the situation during the second stage of labour.
- I was tired during the second stage of labour.
- I have positive memories from the second stage of labour.
- I have negative memories from the second stage of labour.
- I felt vulnerable during the second stage of labour.
- I was afraid during the second stage of labour.
- I was concerned about my child’s health during the second stage of labour.
- The midwife understood my needs during the second stage of labour.
- I felt included in decision about birth position.

**Items rated on a 7-point Likert scale**

- How much of a feeling of being in control did you experience during the second stage of labour? *not in control (1)–completely in control (7*).
- During the second stage of labour I felt: *no pain at all (1)–worst imaginable pain (7*).
- I experienced the pain as: *very negative (1)–very positive (7*).
- How did you experience the length of the second stage of labour? *drawn out (1)–fast (7*).
- When you look back on the birth now, how safe did you feel during the second stage of labour? *very unsafe (1)–totally safe (7*).

The development of the items regarding the second stage of labour was carried out in several steps. We assumed that the intervention would only affect experiences of the second stage, as the second midwife would only be present during the late second stage. A search for validated instruments on women’s experiences of the second stage of labour in PubMed, CINAHL and Google Scholar yielded no results. All existing scales on birth experience report on the overall experience.^21^ Therefore, we carried out a review of the literature^2 3^ and consulted with members of the research group with contemporary clinical expertise before construction of 15 items covering experiences of the second stage of labour. When developing the items, inspiration was drawn from dimensions of the overall birth experience as described in the literature. These dimensions include feelings and perceptions during labour,^22^ own capacity, support and perceived safety.^23^

To test the validity and the relevance of the items in the questionnaire, 10 women who had recently given birth were invited to review the questionnaire using a think-aloud process with cognitive interviewing.^24 25^ The women were recruited at the postnatal ward and postnatally in primary care. Individual interviews were carried out 4–10 weeks after birth. Of the 10 women recruited, 9 women were primiparous, and 1 woman had given birth after a previous caesarean section. All items were reported as relevant and acceptable to the women. However, some minor adjustments were made regarding the wording of the items to improve coherence. The questionnaire was developed in Swedish and then further translated to English in an iterative process, where a bilingual translator did the first translation, followed by discussions in the research group. Amendments were proposed and discussed on several occasions in order to retain the meaning of the items.

### Data collection

The questionnaire was provided electronically or as a postal survey for Swedish-speaking women. The questionnaire in English was available only as a postal survey. To increase participation, four reminders were sent out to non-responders with 1-week intervals between each. The first two reminders were sent as text messages, the third reminder was sent as a postal survey and the fourth reminder was sent as a text message. Since reminders were sent to women up to 2 months after birth, it was decided to include responses up to 4 months after the birth and later responses were excluded.

The analysis consists of data from the follow-up questionnaire, 15 items (box 1). Of these, 10 items were rated on a 4-point Likert scale from 1 (strongly agree) to 4 (disagree) and 5 items used a 7-point scale. The responses on 4-point Likert scales were reversed in order for higher scores to indicate greater agreement with the statements. Data were also collected from case report forms and from each participating unit’s database. Trial data used in this study were: maternal age, ethnicity, pre-pregnancy body mass index, marital status, parity, onset of labour, epidural analgesia, augmentation with oxytocin, duration of the total second stage of labour, time for start of the active second stage, birth position, SPT, fetal birth weight, Apgar score and postpartum haemorrhage. Maternal age was categorised into three groups (<25 years, 25–35 years, >35 years) and postpartum haemorrhage was dichotomised (<500 mL, ≥500 mL).

### Statistical analyses

Data were analysed by intention to treat, that is, in the randomised groups, regardless of deviations from the group allocation. For background variables and labour and birth characteristics, mean and SD were calculated for normally distributed data, whereas median and IQR were used for non-normally distributed data. To compare background variables and labour and birth outcomes between the groups, χ^2^ test was calculated for dichotomous variables and the Student’s t-test for continuous data, with the significance level set at <0.05 (two-tailed) for all analyses. To compare experiences of the second stage of labour between intervention and standard care, the Student’s t-test was used to calculate mean differences (MDs) with 95% CIs and the Mann-Whitney U test was used to calculate p values.

If any statistically significant differences were found for any of the items, a subgroup analysis was conducted where the results for each study site was compared using the Student’s t-test and the Mann-Whitney U test.

All statistical analyses were performed using IBM SPSS software (V.28).

### Patient and public involvement

Women were involved in the design and conduct of this trial by the inclusion of a representative for the women’s perspective in the steering committee. However, they will not participate in disseminating the results to the public.

## Results

Of the 2831 women who gave consent to participate in the follow-up, 2233 (78.9%) responded to the questionnaire and of those, 1937 (86.7%) women responded within 2 months after the birth. A majority of the women responded electronically (68.8%), and 31.2% completed the postal survey. Only 124 women (5.6%), responded to the English version. In total, the overall response rate was 78.5% after exclusion of 12 late responders who submitted the questionnaire later than 4 months after birth (figure 1).

### Responders and non-responders

Women who responded to the questionnaire were significantly older than non-responders (mean 30.0; SD 4.28 vs mean 27.9; SD 4.77; p<0.001), were significantly more likely to be living with a partner compared with non-responders (90.7% vs 80.9%; p<0.001) and were more often of Nordic ethnicity (75.8% vs 40.3%; p<0.001) (online supplemental table S1).

### Background, labour and birth characteristics

Women in the intervention and standard care groups were similar in background characteristics (table 1). More than 60% of the women in both groups used epidural analgesia for pain relief and almost 70% had their labours augmented with oxytocin. Differences between the groups were found in the incidence of SPT and the length of the active second stage of labour. The number of respondents in the follow-up questionnaire who had experienced SPT, reflected the results of the Oneplus trial,^16^ with 3.8% in the intervention group compared with 5.8% in the standard care group (p=0.03) sustaining SPT (table 2). A difference in the median duration of the active second stage of labour also reflected the results of the Oneplus trial: women in the intervention group had a slightly longer duration compared with those allocated to standard care (35.0 min vs 33.0 min, p=0.01). The incidence of Apgar score below 7 was low with no statistical difference between the groups.

**Table 1:** Background characteristics of women responding to the follow-up questionnaire

	Randomised to two midwives (intervention) (n=1127)	Randomised to one midwife (standard care) (n=1094)	P value
Maternal age at birth (mean, SD)	30.0 (4.37)	30.1 (4.19)	0.65
BMI (mean, SD)	24.3 (4.50)	24.6 (4.50)	0.21
Missing	57 (5.1)	50 (4.6)	
Parity			
Nulliparous	1058 (93.9)	1022 (93.4)	0.66
VBAC	69 (6.1)	72 (6.6)	
Marital status			0.46
Married or living with a partner	1015 (90.1)	1000 (91.4)	
Not living with a partner or other life situation	50 (4.4)	42 (3.8)	
Missing	62 (5.5)	52 (4.8)	
Ethnicity			
Nordic	852 (75.6)	832 (76.1)	0.83
European	108 (9.6)	107 (9.8)	0.88
African	18 (1.6)	19 (1.7)	0.80
Middle Eastern	60 (5.3)	62 (5.7)	0.73
South American	17 (1.5)	16 (1.5)	0.93
Asian	65 (5.8)	52 (4.8)	0.28
Missing	7 (0.6)	6 (0.5)	

Data are n (%) or mean (SD). index. Comparisons between groups are calculated using sStudent’s t -test (continuous variables) and χ2 test (dichotomous variables).

BMIbody mass indexVBACvaginal birth after caesarean

**Table 2:** Labour, birth and neonatal outcomes for women responding to the follow-up questionnaire

	Randomised to two midwives (intervention) (n=1127)	Randomised to one midwife (standard care) (n=1094)	P value
Maternal outcomes			
Onset of labour			
Spontaneous	827 (73.4)	804 (73.5)	0.95
Induction	300 (26.6)	290 (26.5)	
Epidural analgesia/spinal	685 (60.8)	702 (64.2)	0.10
Augmentation with oxytocin	781 (69.3)	760 (69.5)	0.93
Birth position			
Lateral	399 (35.4)	402 (36.7)	0.56
Lithotomy/recumbent	404 (35.8)	364 (33.3)	0.18
Sitting	193 (17.1)	210 (19.2)	0.22
Kneeling/standing	51 (4.5)	60 (5.5)	0.31
Birth chair/squatting	43 (3.8)	32 (2.9)	0.24
All four	24 (2.1)	18 (1.6)	0.39
Missing	13 (1.2)	8 (0.7)	
Total second stage of labour—minutes (median, IQR)	104.5 (58.0–169.0)	102.0 (58.0–166.0)	0.62
Missing	3 (0.3)	2 (0.2)	
Active second stage—minutes (median, IQR)	35.0 (24.0–53.0)	33.0 (22.0–49.0)	0.01
Missing	30 (2.7)	27 (2.5)	
Episiotomy	71 (6.3)	64 (5.9)	0.66
Severe perineal trauma	43 (3.8)	63 (5.8)	0.03
Postpartum haemorrhage >500 mL	348 (30.9)	350 (32.0)	
Missing	25 (2.2)	32 (2.9)	0.49
Neonatal outcomes			
Apgar <7 at 5 min	7 (0.6)	10 (0.9)	0.43
Birth weight (mean, SD)	3521 (427.9)	3510 (428.3)	0.53
Missing	1 (0.1)	1 (0.1)	

Data are n (%), median (IQR) or mean (SD). index. Comparisons between groups are calculated using χ2 test (dichotomous variables) and the Mann -Whitney U test (continuous variables).

BMIbody mass index

### Women’s experiences of the second stage of labour

Overall, women’s experiences did not differ between intervention and standard care groups. Women in both groups reported a high mean score for the item ‘The midwife understood my needs during the second stage of labour’ (intervention: mean 3.43; SD 0.79; standard care: mean 3.46; SD 0.81) (table 3, onlinesupplemental figures 1  2). There were no statistically significant differences in women’s experiences concerning feelings of control, vulnerability, pain and memories during the second stage of labour between the two groups. Women allocated to the intervention scored significantly lower on the item ‘I could handle the situation during the second stage of labour’ (mean 3.18, SD 0.87) compared with women allocated to standard care (mean 3.26, SD 0.84) (p=0.03). A subgroup analysis performed for this item showed that the result originated from one of the study sites, where a statistically significant difference between the groups was found (intervention group: mean 3.11, SD 0.90 vs standard care group: mean 3.32, SD 0.78; MD 0.21, 95% CI 0.08 to 0.35, p=0.01). No significant differences were found for the other study sites.

**Table 3:** Experience of the second stage of labour when randomised to two and one midwife according to the follow-up questionnaire in the Oneplus trial

	Randomised to two midwives (intervention) (n=1127)	Randomised to one midwife (standard care) (n=1094)	Mean difference (95% CI)	P value
Items rated on 4-point Likert scale				
I felt strong during the second stage of labour				
Mean (SD)	2.67 (1.01)	2.75 (0.99)	0.08 (0.00 to 0.17)	0.06
I could handle the situation during the second stage of labour				
Mean (SD)	3.18 (0.87)	3.26 (0.84)	0.08 (0.01 to 0.15)	0.03
I was tired during the second stage of labour				
Mean (SD)	2.60 (1.09)	2.56 (1.10)	−0.04 (−0.13 to 0.05)	0.42
I have positive memories from the second stage of labour				
Mean (SD)	2.68 (1.03)	2.69 (1.03)	0.01 (−0.07 to 0.10)	0.79
I have negative memories from the second stage of labour				
Mean (SD)	1.74 (0.92)	1.69 (0.89)	−0.05 (−0.12 to 0.03)	0.20
I felt vulnerable during the second stage of labour				
Mean (SD)	1.39 (0.75)	1.38 (0.73)	−0.01 (−0.07 to 0.06)	0.86
I was afraid during the second stage of labour				
Mean (SD)	1.77 (0.95)	1.73 (0.93)	−0.04 (−0.12 to 0.04)	0.35
I was concerned about my child’s health during the second stage of labour				
Mean (SD)	1.74 (0.94)	1.74 (0.92)	0.00 (−0.08 to 0.07)	0.91
The midwife understood my needs during the second stage of labour				
Mean (SD)	3.43 (0.79)	3.46 (0.81)	0.03 (−0.04 to 0.10)	0.20
I felt included in decision about birth position				
Mean (SD)	2.76 (1.14)	2.81 (1.12)	0.05 (−0.05 to 0.14)	0.30
Items rated on 7-point Likert scale				
How much of a feeling of being in control did you experience during the second stage of labour? *not in control (1)–completely in control (7*)				
Mean (SD)	4.01 (1.78)	4.04 (1.79)	0.03 (−0.12 to 0.18)	0.68
During the second stage of labour I felt *no pain at all (1)–worst imaginable pain (7*)				
Mean (SD)	5.01 (1.67)	4.93 (1.66)	−0.08 (−0.22 to 0.06)	0.13
I experienced the pain as *very negative (1)–very positive (7*)				
Mean (SD)	3.81 (1.71)	3.85 (1.74)	0.04 (−0.10 to 0.19)	0.58
How did you experience the length of the second stage of labour? *drawn out (1)–fast (7*)				
Mean (SD)	4.01 (2.15)	4.10 (2.12)	0.09 (−0.09 to 0.26)	0.41
When you look back at the birth now, how safe did you feel during the second stage of labour? *very unsafe (1)–totally safe (7*)				
Mean (SD)	5.69 (1.65)	5.75 (1.62)	0.06 (−0.07 to 0.20)	0.37

Higher values indicate greater agreement with the statement. Mean differences with 95% CI CI are calculated with sStudent’s t -test. P- values are calculated with the Mann-Whitney U- test.

The 4-point Likert scales range from 1 (strongly agree) to 4 (disagree). The responses were reversed in order for higher scores to indicate greater agreement with the statement. Missing values range between 0.3% and 1.4%.

## Discussion

The findings from this study show that there were no statistically significant differences between the groups regarding women’s experiences of pain, feelings of vulnerability or being in control and experiences of the length of the second stage of labour. However, women randomised to be assisted by two midwives agreed to a lesser extent that they could handle the situation during the second stage of labour. Conducted subgroup analyses revealed that this result originated from one of the study sites.

In this trial, assistance by one or two midwives during the late second stage of labour did not affect women’s experiences of the second stage. This is in line with findings from other trials investigating women’s birth experiences when randomised to an intervention or standard care during labour.^26^,^28^ The questionnaires used to measure birth experience in these trials include similar items as in the present study. The intervention under investigation had little or no impact on women’s birth experiences in these trials. This might be explained by the fact that other factors such as support from caregivers and feelings of being safe and in control and participation in decision-making may be more important for the birth experience.^29 30^

The differences in the incidence of SPT and the length of the second stage of labour between the two groups have been reported earlier, in the results of the Oneplus trial.^16^ In the present study, these differences were not reflected in women’s experiences of the second stage. A prolonged labour has been associated with negative birth experiences.^31^ However, the significant difference in the length of the active second stage in this study appears to be clinically irrelevant to the women’s experiences in this study. SPT has also been associated with negative birth experiences,^6^ which could lead to the assumption that women in the standard care group would rate their experiences more negatively than those in the intervention group. However, our results did not reflect that SPT affected women’s overall experiences of the second stage of labour after birth. A possible explanation for this result is that, although fewer women sustained SPT in the intervention group, the proportion of SPT was relatively small, in the total study population. Furthermore, since this was an unmasked trial and participants were not blinded to their group allocation, this could affect the result.

Women randomised to two midwives scored significantly lower on one item; ‘I could handle the situation during the second stage of labour’. This might signify that the intervention had some negative impact. A possible interpretation of this finding is that an additional caregiver during the second stage of labour may lead to a changed focus from supporting and communicating with the woman, to collegial collaboration with the aim of preventing perineal trauma. Since effective communication with the caregiver during childbirth has been described as an essential component of the experience of care,^32^ it is conceivable that interactions between caregivers in the birthing room may affect their communication with the woman during this stage. Professional support and experience of trusting relationships with caregivers have been described as significant factors for women to be able to cope during childbirth.^33 34^ It is possible that this could not always be accommodated with two midwives present.

However, a recently published study within the Oneplus trial showed that among women actually receiving the intervention, only 6,7% of the women were negative towards being assisted by an additional midwife during the second stage.^35^ The subgroup analysis conducted to understand the differences between the groups showed that only one of the study sites contributed to the difference. This indicates that presumably, it is not the intervention per se that causes the difference, but rather that contextual factors might be at play. Workplace culture and norms are known to influence collaboration between care providers during labour and birth^36^ and workplace conditions may affect attitudes towards practice change.^37^ This may further be reflected in the relationship with the woman and might explain why women’s experiences of being able to handle the situation differed between the study sites. However, as the difference between the groups only applied to one single item, with a small MD in a large study sample, there is a possibility of this being a chance finding.^38^ The significance of this result should therefore be interpreted with caution.

### Strengths and limitations

Strengths of this study include the high response rate to the questionnaire and the randomised design which is considered the most rigorous and robust design for determining a cause and effect between an intervention and an outcome.^39^ The items regarding experiences of the second stage of labour were developed according to existing literature^2 3^ and validated by women in a think-aloud process.^24^ By including the user perspective and involving women in the design of the questionnaire, the items constructed were shown to be comprehensible and acceptable to the women. Furthermore, the questionnaire was provided in both Swedish and English, enabling more women to respond.

This study has several limitations. First, due to the nature of the intervention, it was not possible to blind the participating women or the midwives. Being aware of group allocation may have influenced women’s responses, potentially introducing bias. Second, although the items in the questionnaire were tested for face validity, no further measures were undertaken to validate the items. The use of single items is a limitation as using a psychometrically tested scale would have strengthened the reliability of the results. However, this was not possible as no existing validated scales reported specifically on experiences of the second stage of labour. Third, since validated scales measure latent constructs of attitudes and experiences, the use of single items could introduce measurement errors.^24^ Although the women who participated in the face validation proposed no amendments regarding the item ‘I could handle the situation during the second stage of labour’, this is quite a broad statement which could have different meanings to individual women. Additional items that measured the same latent construct, such as coping with or managing the situation, could have reduced the risk of object-specific measurement errors. Fourth, the non-responders were significantly younger, less often married or cohabiting with their partner and more often of ethnicity other than Nordic. These are factors known to be connected with distrust in healthcare professionals, negative experiences of care and lower response rates in surveys.^40^,^42^ Therefore, our results cannot be generalised to this population. However, the number of non-responders was low and they were equally distributed between the two groups. Fifth, the ideal time point for measuring overall birth experiences has been extensively discussed.^43^,^45^ Measuring the overall birth experience too soon after the birth may be influenced by the immediate relief of giving birth to a healthy child, which may mask other reactions.^44^ The ideal time point for measuring the experience of the second stage of labour is not known and may also vary depending on the research question.

Although we found no differences between women assigned to the intervention and those receiving standard care, a recent study within the Oneplus trial reported that collegial midwifery assistance was particularly appreciated by women with fear of birth, those with lower educational attainment, and those who did not have Swedish as their native language.^35^ This reinforces the findings from the present study, altogether showing that collegial midwifery assistance is a well-accepted intervention that can be safely implemented into standard care to reduce SPT. However, as the trial was conducted in the Swedish setting, the result may not be generalisable to other countries or contexts. To further understand the implication of the intervention on women’s experiences, a qualitative study could provide important insights.

## Conclusion

The findings from this study show that overall women’s experiences of the second stage did not differ between women randomised to one or two midwives. Women randomised to assistance by two midwives agreed to a lesser extent that they could handle the situation during the second stage of labour. However, the MD was small and only significant for one of the study sites. The finding that the intervention does not affect the experience of the second stage is of importance as being assisted by two midwives reduces SPT.

## supplementary material

10.1136/bmjopen-2023-077458 online supplemental file 1

10.1136/bmjopen-2023-077458 online supplemental file 2

10.1136/bmjopen-2023-077458 online supplemental file 3

10.1136/bmjopen-2023-077458 online supplemental file 4

## Data Availability

Data may be obtained from a third party and are not publicly available.
