# Supplementary material for: Impact of collegial midwifery assistance during second stage of labour on women’s experience: a follow-up from the Swedish Oneplus randomised controlled trial
Source: BMJ Open. 2024 Jul 26;14(7):e077458. doi: 10.1136/bmjopen-2023-077458 (PMC11284909; doi:10.1136/bmjopen-2023-077458)
Supplement: online supplemental file 1 [file bmjopen-14-7-s001.pdf]

Supplementary material, Appendix . Items used from the Oneplus follow-up questionnaire

What is your marital status?

☐Married/living with your partner
☐Have a partner but do not live together
☐Single

☐Other...

What is your highest level of education?

☐ Year 1-8
☐ Year 9-12
☐ Completed a higher education certificate or diploma course
☐ Completed a Bachelor’s degree or higher
☐ Other training……………………………..

I felt strong during the second stage of labor
I felt could handle the situation during the second stage of labor
I was tired during the second stage of labor
I have positive memories from the second stage of labor
I have negative memories from the second stage of labor
I felt vulnerable during the second stage of labor

I was afraid during the second stage of labor
I was concerned about my child's health during the second stage of labor

Feelings of worry and fear is common among women facing childbirth. Did you experience fear of giving birth before childbirth?

☐Yes ☐No

If you answered yes to the above question, did you receive any treatment for fear of childbirth?

☐ Yes, counseling and birth planning with a midwife at an Aurora/Irma unit
☐ Yes, counseling and birth planning with a doctor/obstetrician at an Aurora/Irma unit
☐ Yes, counseling with a midwife at the antenatal clinic/mödravårdscentralen ☐
Yes, counseling with a psychologist
☐ Other treatment/support, please describe:
………………………………………………………………………………..
☐ No treatment received

The following questions are statements about your experience of the second stage of labor. The second stage of labor begins when the cervix is completely dilated (open), and ends with the birth of your baby. Please mark the best alternative for you

The midwife understood my needs during the second stage of labor
I felt included in decision about birth position

Response options to all items in the table above:

Strongly agree	Mostly agree	Agree in part	Disagree
☐	☐	☐	☐

How much of a feeling of being in control did you experience during the second stage of labor?

Not in control 1 2 3 4 5 6 7 Completely in control

During the second stage of labor, I felt:

No pain at all 1 2 3 4 5 6 7 Worst imaginable pain

I experienced the pain as

Very negative 1 2 3 4 5 6 7 Very positive

How did you experience the length of the second stage of labor?

Drawn out	1	2	3	4	5	6	7	Fast

When you look back on the birth now, how safe did you feel during the second stage of labour?

Very unsafe	1	2	3	4	5	6	7	Totally safe
