# Supplementary material for: Impact of collegial midwifery assistance during second stage of labour on women’s experience: a follow-up from the Swedish Oneplus randomised controlled trial
Source: BMJ Open. 2024 Jul 26;14(7):e077458. doi: 10.1136/bmjopen-2023-077458 (PMC11284909; doi:10.1136/bmjopen-2023-077458)
Supplement: online supplemental file 2 [file bmjopen-14-7-s002.pdf]

Supplementary material, table S1. Background characteristics for responders and non-responders

Background characteristics	Responders (n=2221)	Non-responders (n=598)	p-value
Maternal age at birth (mean, SD)	30.0 (4.28)	27.9 (4.77)	<0.001
BMI (mean, SD)	24.5 (4.50)	24.9 (5.11)	0.05
Missing	107 (4.8)	37 (6.2)	
Marital status			<0.001
Married or living with a partner	2015 (90.7)	484 (80.9)	
Not living with a partner or other life situation	110 (5.0)	87 (14.5)	
Missing	96 (4.3)	27 (4.5)	
Ethnicity			
Nordic	1684 (75.8)	241 (40.3)	<0.001
European	215 (9.7)	99 (16.6)	<0.001
African	37 (1.7)	47 (7.9)	<0.001
Middle Eastern	122 (5.5)	134 (22.4)	<0.001
South American	33 (1.5)	12 (2.0)	0.35
Asian	117 (5.3)	54 (9.0)	<0.001
Missing data	13 (0.6)	11 (1.8)	

Data are n (%) or mean (SD). BMI = body mass index. Comparisons between groups are calculated using students *t* test (continuous variables) and $\chi 2$ test (dichotomous variables).
