# Supplementary figures and images for: Impact of collegial midwifery assistance during second stage of labour on women’s experience: a follow-up from the Swedish Oneplus randomised controlled trial
Source: BMJ Open. 2024 Jul 26;14(7):e077458. doi: 10.1136/bmjopen-2023-077458 (PMC11284909; doi:10.1136/bmjopen-2023-077458)

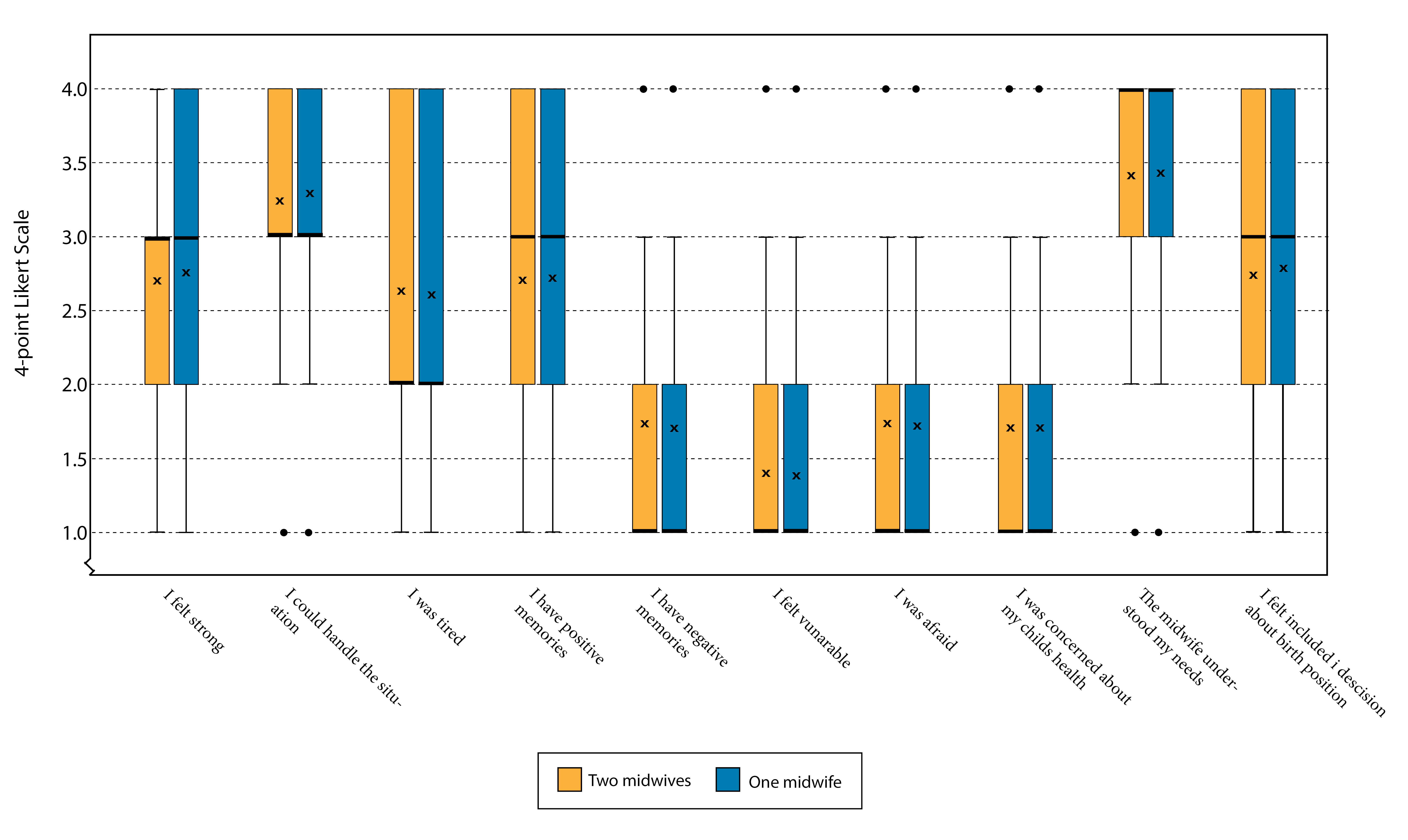

Supplement: online supplemental file 3 [file bmjopen-14-7-s003.jpg]

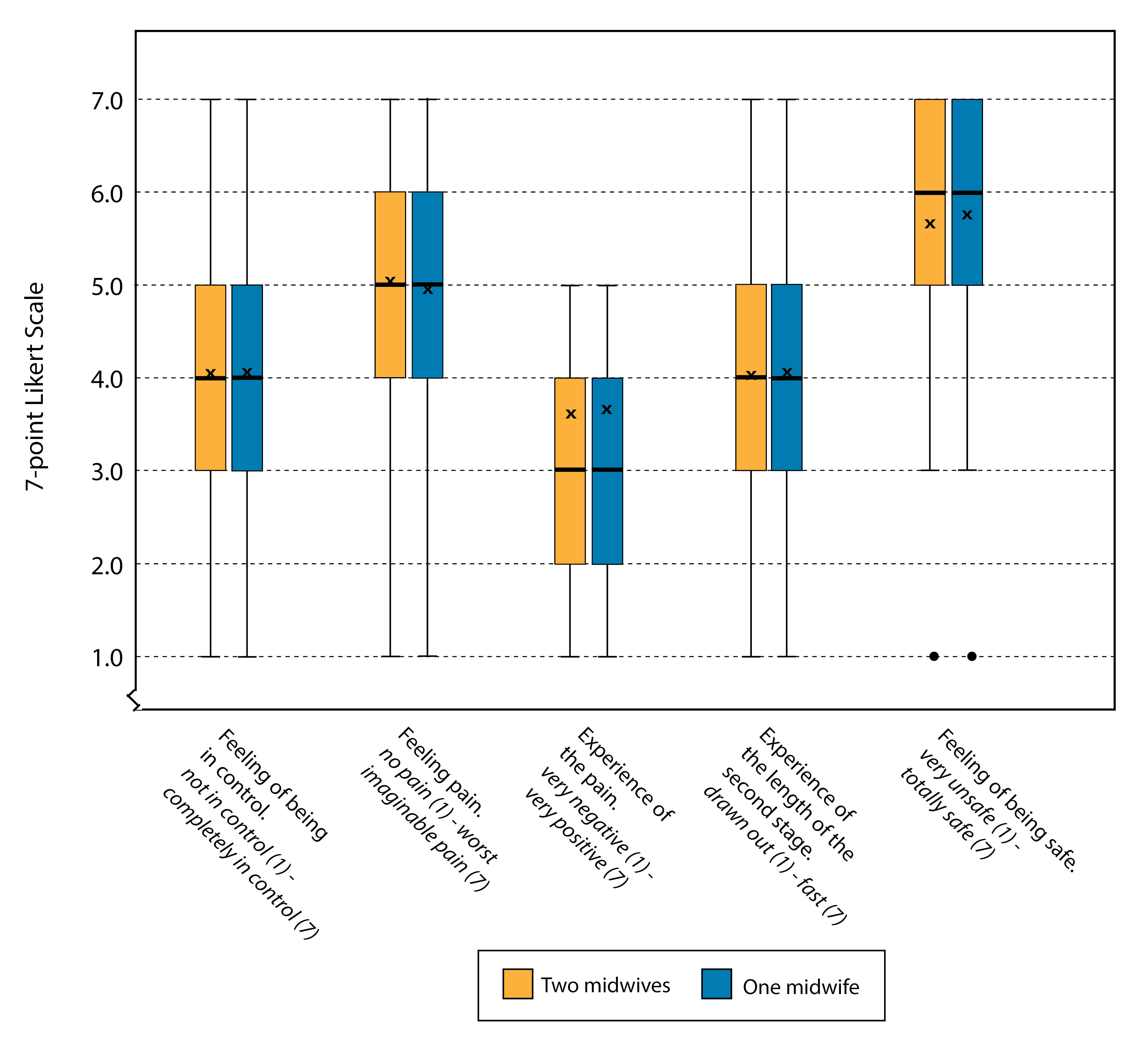

Supplement: online supplemental file 4 [file bmjopen-14-7-s004.jpg]
