# Supplementary material for: Impact of collegial midwifery assistance during second stage of labour on women’s experience: a follow-up from the Swedish Oneplus randomised controlled trial
Source: BMJ Open. 2024 Jul 26;14(7):e077458. doi: 10.1136/bmjopen-2023-077458 (PMC11284909; doi:10.1136/bmjopen-2023-077458)
Supplement: Supplementary file 5 [file bmjopen-2023-077458.reviewer_comments.pdf]

PEER REVIEW HISTORY

BMJ Open publishes all reviews undertaken for accepted manuscripts. Reviewers are asked to complete a checklist review form (**http://bmjopen.bmj.com/site/about/resources/checklist.pdf**) and are provided with free text boxes to elaborate on their assessment. These free text comments are reproduced below.

ARTICLE DETAILS

**TITLE (PROVISIONAL)**	Impact of collegial midwifery assistance during second stage of labour on women's experience: A follow-up from the Swedish Oneplus randomised controlled trial
**AUTHORS**	Häggsgård, Cecilia; Edqvist, Malin; Teleman, Pia; Tern, Helena; Rubertsson, Christine

VERSION 1 – REVIEW

**REVIEWER**	Schwind, Bettina<br>Kalaidos University of Applied Sciences, Careum School of Health
**REVIEW RETURNED**	10-Aug-2023

**GENERAL COMMENTS**	It was a pleasure to review the manuscript entitled “Impact of collecgial midwifery assistance during second stage of labour on women’s experiences: A follow-up study from the Oneplus randomized controlled trial”. The overall aim of the study was to compare the experience of the second stage of labour between women assigned to collegial midwifery and standard care. The study is important because it reports women's experiences of the intervention, which is key to ensuring high quality care and preventing trauma. It also adds well to previously published data from the Oneplus trial.<br><br>Overall, the manuscript is clearly structured and reads well, but I have some suggestions for improvement. In my view, a key aspect is to make more explicit which type of women's experiences and/or which dimensions (control? vulnerability? pain? other?) were assessed for which reasons. This would improve the overall clarity of the manuscript's message and may need to be integrated throughout (methods, results and discussion - see other comments below).<br><br>TITEL<br>I suggest changing the title of the study slightly to make it more precise: “Impact of collegial midwifery support on women's experiences of the second stage of labour”.<br><br>ABSTRACT<br>• I suggest a slight revision of the abstract for clarity - delete the second part "during the second stage of labour" and move the "Oneplus trial" to the methods (p.2 lines 13-17).<br>• Please change the methods accordingly. I would suggest highlighting in the results section that the "handling of the situation" was largely due to a setting.<br>• I would also suggest deleting the first sentence of the conclusion (p.3, line 4), as it is not clear from the manuscript whether the

	measured dimensions necessarily allow the assumption of general satisfaction in both arms.<br>• Strengths and limitations:<br>• Please, specify why the RCT as design is a strength (no reasons are given, also in the discussion section (p.14/line 45). Also, from the methods part, the patient involvement throughout the study (design and implemention) is not made fully clear (see comments in methods). As a limitation; I would suggest to include that there was no masking (midwifes and patients) done/possible which might have introduced bias (especially on women's experiences).<br>• Please state why the RCT design is a strength (no reasons are given, also in the discussion section (p.14/line 45). Patient involvement may need adaption (see comments methods and strength/limitations), there is some inconsistencies across the manuscript. As a limitation, I would suggest including that there was no masking of midwives and patients, which could have introduced bias (especially in women's experiences).<br><br>INTRODUCTION<br>• p. 3 line 40/41 "During the second stage of labour…" might be deleted.<br>• p.5 line 11: I suggest referring directly to the Oneplus study.<br><br>METHODS<br><br>Study design<br>• P.5 lines 41-47: "This study...by a midwife.", repetition of the aims of the present study and the context of the Oneplus trial, so please either delete or move aspects you consider important to the introduction.<br>• Please include a sentence stating that no masking of midwives/women was possible.<br>• P.5, line 7: Is it true that the information material was available in English, Swedish, Arabic and Farsi, but the questionnaire was only available in English and Swedish? If so, please give reasons for this.<br><br>Questionnaire to women one month after birth<br>• p.6. line 34: I suggest changing the subtitle to "questionnaire development"<br>• p.6. line 39/38 and line 44/45: the questionnaire was developed to assess women´s experiences of the second stage of labour and collegial midwifery assistance (intervention), perineal pain, initiation of breastfeeding, and postnatal depression. The wording is a little confusing and needs some clarification, as the study analysed the experiences of the second stage and these are shown in box 1. Please remove the 'experience of collegial midwifery support' (underlined above) as this was probably not part of the questionnaire developed but was intended to be assessed. For the sake of clarity, I will assume that perineal pain refers to the post-partum period. A number of items in box 1 ask about the experience of pain. Therefore, I suggest that the sentence be slightly revised to help the reader to follow more quickly: e.g. "Experiences of the second stage of labour (intervention experiences) and perineal pain...(postpartum experiences)".<br>• p.6. line 55 to p.7 line 16, including box 1: Please describe in more detail how the questionnaire was developed: The aim of the study was to assess women's experiences of the second stage of labour. However, the results of the literature review (key

	dimensions of women's experiences) remain unclear, and which dimensions have been identified or added by experts as relevant to the second stage? And how did these processes contribute to the items in Box 1? Specifically, which item contributes to which dimension/attribute of women's experiences that you have identified as relevant for the second stage? Which I assume from reading the results/conclusion are 'control', 'vulnerability' and 'pain', but also others. Please explain the development of the questionnaire in the text, but also add the overall dimensions in box 1, as this would help the reader to better understand your results.<br>• Another aspect that needs to be clarified in relation to the development of the questionnaire is the 'involvement' of women, as compared to the 'patient and public involvement' section (p.8, line 59 - p.8, line 4): Here you mention that women were involved in the development of the questionnaire, but this is not mentioned and explained in the methods section. This needs to be included in the development of the questionnaire and/or excluded from the PPI section, as well as the strengths/limitations. Could you also add some information about the women? For example, did they receive collegial midwifery support? How many children were born? And how were they recruited/sampled?<br>• Box 1: Please add the dimensions of experience and allocate the corresponding items.<br>• This section does not report on a pre-test of the questionnaire. If this is the case, you may want to add this to the limitations; and/or give reasons why this was not necessary?<br>•<br>Data collection<br>• It is not entirely clear who received how many reminders and how (postal / online / SMS?) There seem to be differences between the English and Swedish language groups; you can also specify when you send reminders (one week after...).<br><br>Patient and public involvement<br>• See comment in methods section: If the ten women were not involved in the design of the questionnaire, but only in the validation of the questionnaire, this cannot be considered PPI. Similarly, dissemination of knowledge per se is not considered PPI unless the patients/public are actively involved in the dissemination process and have a say in writing, dissemination and deciding what is relevant to other women. So please consider clarifying or revising.<br><br>RESULTS<br><br>Background and labour birth characteristics<br>• P9 line 47: You compare the current results to those of the Oneplus trial, please provide the reference here.<br><br>Women's experiences of the second stage of labour<br>• P. 11 line 54/55: You report the results of the questionnaire, which you may (slightly) revise after revising the methods/box 1, including dimensions, e.g. also be precise - e.g. you use different wording throughout the manuscript (e.g. feelings, memories, experiences) and also, you do not mention vulnerability here, but in the summary of the discussion you do.<br><br><br>DISCUSSION

	• P. 14 line 12-21 (summary): I would suggest not summarising the results as positive experiences, as this cannot be fully assumed from the results. I suggest staying with "The results of the study show no significant differences...". I also suggest adding that the differences found in handling the situation were due to one side of the study (see commentary abstract).<br>• P 14. Line 23-30: Please be specific, add and elaborate, as the comparison with the existing RCTs is a bit short, e.g. the RCTs had very different interventions? What kind of women's experiences were assessed? Can you really compare it with yours? There are also RCTs that have had a positive impact on women's experiences (e.g. https://doi.org/10.1007/s00404-023-07115-4).<br>• P14, line 33-57: Another reason may be that the second stage of labour is relatively short compared to the total length of labour; continuity of care and the relationship with the midwife, but also communication (MIMA model) are also key to women's experience of labour. Such aspects could be further reflected here, also in that the second midwife may not have been perceived as an 'interruption' (understood my needs) and thus changed the overall experience.<br>• Overall, I suggest adding a section on implications for clinical practice and research/unanswered questions, which has not been reflected in the discussion so far.<br>•<br>Strengths and limitations<br><br>• P15 line 44: Why is the RCT considered a strength, please include a short explanation.<br>• P15 line 50: The involvement of women in the design of the questionnaire is not described in the methods. Please, revise accordingly.<br>• P15 line 59: Midwives were also aware, right? Please add to the bias.<br>• Please add to the limitations that the questionnaire was not pre-tested.<br>• P16 line 16-18: In order to better understand women's experiences, a follow-up qualitative study could have been added to better understand the wider implications in more depth, as in the dimensions of 'coping with the situation', which you may want to add as a limitation, also in terms of what collegial midwife means or changes in women's experiences.<br><br>CONCLUSION<br>As mentioned above, I would recommend not to report 'positive' experiences, but to stay with the findings as in differences/similarities found, and to report the respective dimensions, and to conclude from this the implications for practice here (see discussion section).

**REVIEWER**	Purno, Nabila H.<br>United Nations Population Fund
**REVIEW RETURNED**	20-Aug-2023

**GENERAL COMMENTS**	This is a very clear and well written article- congratulations to the author for researching on this topic and preparing the manuscript. Some minor comments: in the introduction, to describe usual causes of SPT, a bit on how women's experience in the second

	stage of labor can contribute to SPT. The authors can also describe a little bit about collegial midwifery assistance and what is the theoretical basis for them to assume that two midwives should improve or impact labour experiences and SPT.<br><br>In the results section, it would have been good to have some reference to neonatal birth outcomes - since that may also have introduced recall bias.<br><br>In the discussion section,<br>Other than these, the paper is very clear and articulate.

**REVIEWER**	Oscarsson, Marie<br>Linnaeus University, Department of Health and Caring Sciences
**REVIEW RETURNED**	22-Oct-2023

**GENERAL COMMENTS**	Congratulations for your large work – the subject of the study is of interest especially in midwifery practice. I have only one suggestion-Please, do not repeat the results from tables. This section would be more fluent to read.

**REVIEWER**	Hcini, Najeh<br>West French Guiana Hospital Center
**REVIEW RETURNED**	14-Jan-2024

**GENERAL COMMENTS**	This is an interesting study in obstetrics, and an experience to be shared with perinatal care providers. This paper merits revision before publication, especially as regards the methodology and the inclusion/exclusion process. Flowchart should be revised.<br><br>Inclusion: 'opting for their first vaginal birth'; exclusion: 'planned cesarean section.' The authors don't mention patients undergoing emergency cesarean section and assisted vaginal delivery.<br><br>Abstract<br><br>Participants: Please provide information on the inclusion or exclusion criteria for the induction of labor. If a cesarean is performed, please provide more precision. Of the 3059 women who gave birth spontaneously, 2831 women consented to participate in the follow-up questionnaire. Please clarify the term 'spontaneously.'<br><br>Inclusion criteria were women opting for their first vaginal birth from gestational week 37+0 with a singleton pregnancy and a live fetus in the vertex presentation.<br><br>Are patients undergoing cesarean section and assisted delivery excluded? I do not see these patients in the flowchart. Please explain.<br><br>Conclusions:<br><br>"Women in both groups were generally satisfied with their experiences of the second stage." Please revise. I think better give only percentage.

	The conclusion should be revised to align with the objective of the study.<br><br>STRENGTHS AND LIMITATIONS OF THIS STUDY:<br><br>This section must be revised to focus on the study's subject. I suggest retaining only the first sentence and changing the others.<br><br>Introduction: More precision must be given on the role of the second midwife. Why do the authors think it can improve the delivery experience?<br><br>Methods:<br><br>Inclusion criteria were women aged between 18 to 47 who opted for them. Is this the inclusion criteria for results? Why 47 years and not 48? I think the authors are confusing this with the study's results.<br><br>"Further inclusion criteria were language proficiency in Swedish, English, Arabic, or Farsi, as study information was only available in these languages," and "questionnaire one month after birth, which was available in Swedish and English." This sentence is confusing, as the principal objective of this second analysis of data is based on the questionnaire. Parturient speaking Arabic and Farsi agreed to participate but received questionnaires in non-adapted languages. I think it's better to exclude these patients from the analysis to avoid confusion.<br><br>Results: Of the 2831 women who consented to participate in the follow-up, 2233 (78.9%) responded to the questionnaire. Here, patients consented to participate but received non-adapted questionnaires, and I think they should be excluded.<br><br>Discussion: "The findings from this study show that women, regardless of randomization, reported overall positive experiences of the second stage of labor." Please revise this sentence as it does not align with the study's objective. "The differences in the incidence of SPT and the length of the second stage of labor between the two groups have been reported earlier, in the results of the Oneplus trial." Please revise this sentence, as a 2-minute difference in the second stage of labor, despite being statistically significant, has limited practical benefit. "A prolonged labor has been associated with negative birth experiences" - where?<br><br>"However, the significant difference in the length of the active second stage in this study appears to be clinically irrelevant for the women’s experiences in this study." Please be cautious in interpreting the results. Do the authors believe that 2 or 3 minutes can change the patient's perception?<br><br>Conclusion:<br><br>"The findings from this study show that women reported overall positive experiences of the second stage of labor." Please provide a conclusion and remove the phrase "regardless of randomization." Include "I could handle the situation during the second stage of labor" as it is significant.

	"as being assisted by two midwives reduces SPT» refers to the results of the same study already published. This information needs to be confirmed by other studies and adapted to the context of application.<br><br>Flowchart:<br><br>Participants were excluded because "27 did not master the Swedish or English language." These patients must be excluded based on the inclusion criteria of this analysis?<br><br>patients undergoing cesarean section and assisted delivery excluded. Are they retained in the analysis?<br><br>RCT: give details of this abbreviation.

**REVIEWER**	Nelson, Sybil Prince<br>Washington and Lee University
**REVIEW RETURNED**	16-Feb-2024

**GENERAL COMMENTS**	To Whom it May Concern,<br><br>I have had the opportunity to review the manuscript titled “Impact of collegial midwifery assistance during the second stage of labour on women’s experience: A follow-up study from the Oneplus randomised controlled trial” submitted to BMJ Open, and I am pleased to share my evaluation. The article is commendably structured, offering a clear motivation, methodology, results, and conclusions. The subject matter is both interesting and significant, addressing an essential aspect of maternal care.<br>The study's design is noteworthy, effectively navigating the inherent limitations of non-blinding due to the intervention method. Despite these constraints, the research provides valuable insights into women's experiences during the second stage of labor. The manuscript adeptly acknowledges potential biases and the challenges of capturing complex attitudes with single-item measurements.<br>A key finding of the study, the statistical difference in severe perineal trauma between the intervention and control groups. Although this finding is marginally significant, it remains an interesting aspect of the study. This outcome, suggesting fewer instances of severe perineal trauma in the midwife-assisted group, merits further emphasis within the manuscript. Highlighting this finding could enhance the paper's contribution to the field, especially given the scarcity of other differing outcomes between the compared groups.<br>However, my primary concern lies with the analysis of Likert scale data. The current approach could be refined to strengthen the manuscript's methodological rigor. I recommend a more nuanced evaluation of the Likert data, incorporating strategies such as utilizing median and interquartile range (IQR) for capturing central tendency and variability, employing non-parametric tests like the Mann-Whitney or Wilcoxon for comparisons, and considering ordinal logistic regression for modeling relationships with ordinal responses. Additionally, conducting factor analysis and calculating Cronbach’s alpha could further validate the scale's reliability and dimensionality.<br>Implementing these suggestions could significantly enhance the paper's analytical depth, offering a more robust understanding of women's experiences during labor. Not all of these methods are

	necessary, but at least one or two would be beneficial. This, in turn, would bolster the paper's overall strength and its contribution to the existing body of knowledge in maternal healthcare.<br>Thank you for the opportunity to review this manuscript. I look forward to seeing the revised version and believe that with these adjustments, the paper will make a valuable addition to BMJ Open.<br><br>Best regards,<br><br>Sybil Prince Nelson

VERSION 1 – AUTHOR RESPONSE

Reviewer: 1
Dr. Bettina Schwind, Kalaidos University of Applied Sciences, Swiss Tropical and Public Health Institute, University of Zurich
Comments to the Author:
It was a pleasure to review the manuscript entitled "Impact of collecgial midwifery assistance during second stage of labour on women's experiences: A follow-up study from the Oneplus randomized controlled trial". The overall aim of the study was to compare the experience of the second stage of labour between women assigned to collegial midwifery and standard care. The study is important because it reports women's experiences of the intervention, which is key to ensuring high quality care and preventing trauma. It also adds well to previously published data from the Oneplus trial.

Author's reply: Thank you. We are grateful for your time in reviewing the article and for your positive feedback.

Overall, the manuscript is clearly structured and reads well, but I have some suggestions for improvement. In my view, a key aspect is to make more explicit which type of women's experiences and/or which dimensions (control? vulnerability? pain? other?) were assessed for which reasons. This would improve the overall clarity of the manuscript's message and may need to be integrated throughout (methods, results and discussion - see other comments below).

Author's reply: Thank you for your comment. The items are based on factors that previously has been identified as being of significance for the birth experience and/or the experience of the second stage of labour, such as the woman's feelings of safety and of being in control. This was done by carrying out a review of the literature and by discussions within the research group with contemporary clinical expertise, which is described on p. 5, line 5-10.

TITEL
I suggest changing the title of the study slightly to make it more precise: "Impact of collegial midwifery support on women's experiences of the second stage of labour".

Author's reply: Thank you for your suggestion. The term assistance has been used in several articles[1-5] regarding the intervention, including the study protocol, why we have decided to keep the term assistance in the title and throughout the manuscript.

1. Edqvist M, Dahlen HG, Häggsgård C, Tern H, Ängeby K, Tegerstedt G, Teleman P, Ajne G, Rubertsson C. One Plus One Equals Two-will that do? A trial protocol for a Swedish multicentre randomised controlled trial to evaluate a clinical practice to reduce severe perineal

trauma {1}. Trials. 2020 Nov 23;21(1):945. doi: 10.1186/s13063-020-04837-7. Erratum in: Trials. 2020 Dec 2;21(1):993. PMID: 33225972; PMCID: PMC7682019.

2. Edqvist M, Dahlen HG, Häggsgård C, Tern H, Ängeby K, Teleman P, Ajne G, Rubertsson C. The effect of two midwives during the second stage of labour to reduce severe perineal trauma (Oneplus): a multicentre, randomised controlled trial in Sweden. Lancet. 2022 Mar 26;399(10331):1242-1253. doi: 10.1016/S0140-6736(22)00188-X. Epub 2022 Mar 15. PMID: 35303474.

3. Tern H, Edqvist M, Ekelin M, Dahlen HG, Rubertsson C. Swedish midwives' experiences of collegial midwifery assistance during the second stage of labour: A qualitative study. Women Birth. 2023 Feb;36(1):72-79. doi: 10.1016/j.wombi.2022.03.003. Epub 2022 Mar 17. PMID: 35307300.

4. Tern H, Edqvist M, Ekelin M, Dahlen HG, Rubertsson C. Primary midwives' experiences of collegial midwifery assistance during the active second stage of labor: Data from the Oneplus trial. Birth. 2023 Dec;50(4):868-876. doi: 10.1111/birt.12739. Epub 2023 Jul 4. PMID: 37401365.

5. Tern H, Rubertsson C, Ekelin M, Dahlen HG, Häggsgård C, Edqvist M. Women's experiences of being assisted by two midwives during the active second stage of labour: Secondary outcomes from the Oneplus trial. Sex Reprod Healthc. 2024 Mar;39:100926. doi: 10.1016/j.srhc.2023.100926. Epub 2023 Nov 24. PMID: 38041929.

ABSTRACT
• I suggest a slight revision of the abstract for clarity - delete the second part "during the second stage of labour" and move the "Oneplus trial" to the methods (p.2 lines 13-17).

Author's reply: Thank you, we have changed this in the abstract.

• Please change the methods accordingly. I would suggest highlighting in the results section that the "handling of the situation" was largely due to a setting.

Author's reply: Thank you for your comment. We have changed the Design section in the abstract, p1, line 9-10, which now reeds:

Design Analysis of a secondary outcome within the Swedish Oneplus multicenter randomised trial.
We have also added the following text in the results section in the abstract p 1, line 29-30:

Conducted sub-group analyses revealed that this result originated from one of the study sites.

• I would also suggest deleting the first sentence of the conclusion (p.3, line 4), as it is not clear from the manuscript whether the measured dimensions necessarily allow the assumption of general satisfaction in both arms.

Author's reply: Thank you for pointing this out. We agree and have deleted this sentence.

Strengths and limitations:
• Please, specify why the RCT as design is a strength (no reasons are given, also in the discussion section (p.14/line 45).

Author's reply: In the strength and limitation section after the abstract, the first bulletpoint, p 2, line 8-9, now reads:

The randomised design is considered the most rigorous and robust design for determining cause and effect.

We have also specified this in the discussion section on p 15, line 3-4

Strengths of this study include the high response rate to the questionnaire and the randomised design[36] which is considered the most rigorous and robust design for determining a cause and effect.[37]

Also, from the methods part, the patient involvement throughout the study (design and implemention) is not made fully clear (see comments in methods).

As a limitation; I would suggest to include that there was no masking (midwifes and patients) done/possible which might have introduced bias (especially on women's experiences).

Author's reply: Thank you for this suggestion. We have added the following bulletpoint, p 2, line 12-13:

It was not possible to blind the participants or midwives due to the nature of the intervention which may have influenced women's responses.

INTRODUCTION
• p. 3 line 40/41 "During the second stage of labour…" might be deleted.
Author's reply: Thank you for your comment, the sentence is changed according to your suggestion.

• p.5 line 11: I suggest referring directly to the Oneplus study.

Author's reply: We have referred to the Oneplus trial on p. 4, line 12, and changed the sentence:

Further inclusion criteria in the Oneplus trial were language proficiency in Swedish, English, Arabic or Farsi as study information was only available in these languages.

METHODS

Study design
• P.5 lines 41-47: "This study...by a midwife.", repetition of the aims of the present study and the context of the Oneplus trial, so please either delete or move aspects you consider important to the introduction.

Author's reply: Thank you for observing this. We have deleted the two sentences.

• Please include a sentence stating that no masking of midwives/women was possible.

Author's reply: Thank you for pointing this out. We have now added a sentence about blinding on p 15, line 10-11, that reads:

Firstly, due to the nature of the intervention, it was not possible to blind the participating women or the midwives.

• P.5, line 7: Is it true that the information material was available in English, Swedish, Arabic and Farsi, but the questionnaire was only available in English and Swedish? If so, please give reasons for this.

Author's reply: It is correct that information was available in the four languages but that the questionnaire only was available in English and Swedish. Our intention was to translate the questionnaire to all four languages, however, for pragmatic reasons and limited funding this was not possible. We have added a sentence to clarify this, p 4, line 14-15:

As the questionnaire was only available in Swedish and English, hence, women who did not master Swedish or English were not included in the current study.

Questionnaire to women one month after birth
• p.6. line 34: I suggest changing the subtitle to "Questionnaire development"

Author's reply: Thank you, we have changed the subtitle according to your suggestion.

• p.6. line 39/38 and line 44/45: the questionnaire was developed to assess women´s experiences of the second stage of labour and collegial midwifery assistance (intervention), perineal pain, initiation of breastfeeding, and postnatal depression. The wording is a little confusing and needs some clarification, as the study analysed the experiences of the second stage and these are shown in box 1. Please remove the 'experience of collegial midwifery support' (underlined above) as this was probably not part of the questionnaire developed but was intended to be assessed. For the sake of clarity, I will assume that perineal pain refers to the post-partum period. A number of items in box 1 ask about the experience of pain. Therefore, I suggest that the sentence be slightly revised to help the reader to follow more quickly: e.g. "Experiences of the second stage of labour (intervention experiences) and perineal pain...(postpartum experiences)".

Author's reply: Thank you for your suggestion. We have changed the sentences on p 4, line 32 – p 5, line 2, for clarity:

A study specific questionnaire was developed to assess secondary outcomes in the trial; experiences of the second stage of labour and collegial midwifery assistance. Furthermore, questions regarding sociodemographic background and history of physical and mental health. For the purpose of the present study, the items regarding experiences of the second stage were analysed (Box 1).

• p.6. line 55 to p.7 line 16, including box 1: Please describe in more detail how the questionnaire was developed: The aim of the study was to assess women's experiences of the second stage of labour. However, the results of the literature review (key dimensions of women's experiences) remain unclear, and which dimensions have been identified or added by experts as relevant to the second stage? And how did these processes contribute to the items in Box 1? Specifically, which item contributes to which dimension/attribute of women's experiences that you have identified as relevant for the second stage? Which I assume from reading the results/conclusion are 'control', 'vulnerability' and 'pain', but also others. Please explain the development of the questionnaire in the text, but also add the overall dimensions in box 1, as this would help the reader to better understand your results.

Author's reply: Thank you for your comment. The items are based on factors that previously has been identified as being of significance for the birth experience and/or the experience of the second stage of labour, such as the womans' feelings of safety and of being in control. This was done by

carrying out a review of the literature and by discussions within the research group with contemporary clinical expertise, which is described on p. 5, line 5-10. We agree that it would be of value to add dimensions. However, in this study psychometric testing was not performed to identify dimensions and suggest this to be carried out in future research.

• Another aspect that needs to be clarified in relation to the development of the questionnaire is the 'involvement' of women, as compared to the 'patient and public involvement' section (p.8, line 59 - p.8, line 4): Here you mention that women were involved in the development of the questionnaire, but this is not mentioned and explained in the methods section. This needs to be included in the development of the questionnaire and/or excluded from the PPI section, as well as the strengths/limitations. Could you also add some information about the women? For example, did they receive collegial midwifery support? How many children were born? And how were they recruited/sampled?

Author's reply: In the methods section, p 5, line 10-13, we have described that ten women who had recently given birth reviewed the questionnaire using a think aloud process with cognitive interviewing. We have added a sentence on p 5, line 13-16, to include more information about the women:

The women were recruited at the postnatal ward and postnatally in primary care. Individual interviews were carried out 4-10 weeks after birth. Of the ten women recruited, nine women were primiparous, and one woman had given birth after a previous caesarean section.

• Box 1: Please add the dimensions of experience and allocate the corresponding items.

Author's reply: Thank you for your suggestion. However, in this study we have used single items. As described under strengths and limitations, using a psychometrically tested scale could have strengthened the result but this was not possible since no scale report specifically on experiences of the second stage of labour.

• This section does not report on a pre-test of the questionnaire. If this is the case, you may want to add this to the limitations; and/or give reasons why this was not necessary?

Author's reply: Thank you for addressing this. We have added the following text under the strength and limitations section, p 15, line 12-14:

Secondly, although the items in the questionnaire were tested for face validity, no further measures were undertaken to validate the items.

Data collection
• It is not entirely clear who received how many reminders and how (postal / online / SMS?) There seem to be differences between the English and Swedish language groups; you can also specify when you send reminders (one week after...).

Author's reply: On p 6, line 9-12 we have now clarified how many reminders were sent, when they were sent and how:

To increase participation, four reminders were sent out to non-responders with one-week intervals between each. The first two reminders were sent as text messages, the third reminder was sent as a postal survey and the fourth reminder was sent as a text message.

Patient and public involvement
• See comment in methods section: If the ten women were not involved in the design of the

questionnaire, but only in the validation of the questionnaire, this cannot be considered PPI. Similarly, dissemination of knowledge per se is not considered PPI unless the patients/public are actively involved in the dissemination process and have a say in writing, dissemination and deciding what is relevant to other women. So please consider clarifying or revising.

Author's reply: Thank you for your comment. We have deleted "and by including ten women in developing and validating the follow-up questionnaire" in the PPI section. Furthermore, we have now stated that women will not be included in the dissemination process on p. 7, line 27-28:

However, they will not participate in disseminating the results to the public.

RESULTS

Background and labour birth characteristics
• P9 line 47: You compare the current results to those of the Oneplus trial, please provide the reference here.

Author's reply: Thank you for observing this. The reference has now been added. P 8, line 22.

Women's experiences of the second stage of labour
• P. 11 line 54/55: You report the results of the questionnaire, which you may (slightly) revise after revising the methods/box 1, including dimensions, e.g. also be precise - e.g. you use different wording throughout the manuscript (e.g. feelings, memories, experiences) and also, you do not mention vulnerability here, but in the summary of the discussion you do.

Author's reply: Thank you for observing this. We have added "vulnerability" to the sentence on p 10, line 10:

There were no statistically significant differences in women's experiences concerning feelings of control, vulnerability, pain, and memories during the second stage of labour between the two groups.

The wording refers to the items which include feelings (i.e. *I felt strong during the second stage of labor*), memories (i.e. *I have positive memories from the second stage of labor*) and experiences (i.e. *How did you experience the length of the second stage of labor?*)

DISCUSSION
• P. 14 line 12-21 (summary): I would suggest not summarising the results as positive experiences, as this cannot be fully assumed from the results. I suggest staying with "The results of the study show no significant differences...". I also suggest adding that the differences found in handling the situation were due to one side of the study (see commentary abstract).

Author's reply: Thank you for your suggestion. The sentence on p. 13, line 3-5 has been changed and now reads:

The findings from this study show that there were no statistically significant differences between the groups regarding women's experiences of pain, feelings of vulnerability or being in control and experiences of the length second stage of labour.

Furthermore, we have added the following wording to the sentence on p. 13, line 8-9.

However, women randomised to assistance by two midwives agreed to a lesser extent that they could handle the situation during the second stage of labour. Conducted sub-group analyses revealed that this result originated from one of the study sites.

• P 14. Line 23-30: Please be specific, add and elaborate, as the comparison with the existing RCTs is a bit short, e.g. the RCTs had very different interventions? What kind of women's experiences were assessed? Can you really compare it with yours? There are also RCTs that have had a positive impact on women's experiences (e.g. https://doi.org/10.1007/s00404-023-07115-4).

Author's reply: Thank you for your comment. We have added two sentences on p 13, r 13-18 to further elaborate the comparison with the RCTs.

The questionnaires used to measure birth experience in these trials include similar items as in the present study. In those trials The intervention under investigation had little or no impact on women's birth experiences in these trials. This might be explained by the fact that other factors such as support from caregivers and feelings of being safe and in control and participation in decision-making may be more important for the birth experience.[27,28]

• P14, line 33-57: Another reason may be that the second stage of labour is relatively short compared to the total length of labour; continuity of care and the relationship with the midwife, but also communication (MIMA model) are also key to women's experience of labour. Such aspects could be further reflected here, also in that the second midwife may not have been perceived as an 'interruption' (understood my needs) and thus changed the overall experience.

Author's reply: Thank you for this suggestion. On p 14, line 18-12, we have discussed the significance of effective communication for the birth experience and referred to a study by Tern et al (2024) showing that few women felt disrupted by the second midwife.

However, a recent published study within the Oneplus trial showed that among women actually receiving the intervention, only 6,7% of the women were negative towards being assisted by an additional midwife during the second stage.[33]

• Overall, I suggest adding a section on implications for clinical practice and research/unanswered questions, which has not been reflected in the discussion so far.

Author's reply: Thank you for pointing this out. On p 16, line 1-7, we have reflected on the implications for clinical practice:

Although we found no differences between women assigned to the intervention and those receiving standard care, a recent study within the Oneplus trial reported that collegial midwifery assistance particularly appreciated by women with fear of birth, those with lower educational attainment, and those who did not have Swedish as their native language.[33] This reinforces the findings from the present study, altogether showing that collegial midwifery assistance is a well-accepted intervention that can be safely implemented into standard care to reduce SPT.

Strengths and limitations

• P15 line 44: Why is the RCT considered a strength, please include a short explanation.

Author's reply: We have added an explanation on p 15, line 2-4, that reads:

Strengths of this study include the high response rate to the questionnaire and the randomised design[36] which is considered the most rigorous and robust design for determining a cause and effect.[37]

• P15 line 50: The involvement of women in the design of the questionnaire is not described in the methods. Please, revise accordingly.

Author's reply: The involvement of women in the design of the questionnaire is described in the methods section, p 5, line 10-16.

To test the validity and the relevance of the items in the questionnaire, ten women who had recently given birth were invited to review the questionnaire using a think aloud process with cognitive interviewing.[22,23] The women were recruited at the postnatal ward and postnatally in primary care. Individual interviews were carried out 4-10 weeks after birth. Of the ten women recruited, nine women were primiparous, and one woman had given birth after a previous caesarean section.

• P15 line 59: Midwives were also aware, right? Please add to the bias.

Author's reply: Thank you for observing this. The sentence on p. 15, line 10-11. is changed and reads:

Firstly, due to the nature of the intervention, it was not possible to blind the participating women or the midwives.

• Please add to the limitations that the questionnaire was not pre-tested.

Author's reply: Thank you for your suggestion. We have now added to the limitations that no further measures were undertaken to validate the items. P 15, line 12-16.

Secondly, although the items in the questionnaire were tested for face validity, no further measures were undertaken to validate the items.

• P16 line 16-18: In order to better understand women's experiences, a follow-up qualitative study could have been added to better understand the wider implications in more depth, as in the dimensions of 'coping with the situation', which you may want to add as a limitation, also in terms of what collegial midwife means or changes in women's experiences.

Author's reply: We agree that a qualitative study can add a deeper understanding and have added this on p. 16, line 8-9.

To further understand the implication of the intervention on women's experiences, a qualitative study could provide important insights.

CONCLUSION
As mentioned above, I would recommend not to report 'positive' experiences, but to stay with the findings as in differences/similarities found, and to report the respective dimensions, and to conclude from this the implications for practice here (see discussion section).

Author's reply: Thank you. The conclusion has been changed according to your suggestion.

Reviewer: 2
Dr. Nabila H. Purno, United Nations Population Fund

Comments to the Author:
This is a very clear and well written article- congratulations to the author for researching on this topic and preparing the manuscript.

Author's reply: Thank you. We are grateful for your time in reviewing the article and for your positive feedback.

Some minor comments: in the introduction, to describe usual causes of SPT, a bit on how women's experience in the second stage of labor can contribute to SPT.

Author's reply: Thank you for your suggestion. We have added causes to SPT on p 3, line 1-2.

Risk factors for SPT include nulliparity, first vaginal birth after caesarean section, instrumental birth, increased fetal birthweight, increased maternal age and Asian ethnicity.[10,11]

The authors can also describe a little bit about collegial midwifery assistance and what is the theoretical basis for them to assume that two midwives should improve or impact labour experiences and SPT.

Author's reply: Thank you. We have added the following sentence regarding collegial midwifery assistance on p 3, line 19-21:

The midwives were instructed to use the established prevention models[17,18] at their respective labour ward and for the second midwife to be ready to assist the primary midwife if asked and to support the birthing woman if needed.[16]

However, we did not assume that the intervention should improve the experience of the second stage of labour, rather that it can be negatively affected. We have clarified this on p 3, line 21-24:

Since previous research shows that it can be difficult to establish new relationships during the second stage[1], it can be assumed that the assistance of a second midwife may influence the women´s experiences of the second stage negatively.

In the results section, it would have been good to have some reference to neonatal birth outcomes - since that may also have introduced recall bias.

Author's reply: There were no differences in the neonatal outcomes (Table 2). We have added a sentence in the result on p 8, line 27-28:

The incidence of Apgar score below 7 was low with no statistical difference between the groups.

In the discussion section,
Other than these, the paper is very clear and articulate.

Reviewer: 3
Dr. Marie Oscarsson, Linnaeus University
Comments to the Author:
Congratulations for your large work – the subject of the study is of interest especially in midwifery

practice. I have only one suggestion-Please, do not repeat the results from tables. This section would be more fluent to read.

Author's reply: Thank you for your comment and your positive feedback. We have deleted results from the tables that were duplicated in the text and hope it is more fluent to read now.

Reviewer: 4
Dr. Najeh Hcini, West French Guiana Hospital Center
Comments to the Author:
see attached file

This is an interesting study in obstetrics, and an experience to be shared with perinatal care providers.

Author's reply: We are grateful for your feedback and for your time in reviewing the article.

This paper merits revision before publication, especially as regards the methodology and the inclusion/exclusion process. Flowchart should be revised.
Inclusion: 'opting for their first vaginal birth'; exclusion: 'planned cesarean section.' The authors don't mention patients undergoing emergency cesarean section and assisted vaginal delivery.

Author's reply: Thank you for your comment. We have referred to the Oneplus trial on p 4, line 10, for full details.

Abstract
Participants: Please provide information on the inclusion or exclusion criteria for the induction of labor. If a cesarean is performed, please provide more precision. Of the 3059 women who gave birth spontaneously, 2831 women consented to participate in the follow-up questionnaire. Please clarify the term 'spontaneously.'

Author's reply: Thank you for your suggestion. We have clarified the term spontaneously on p 1, line 16-17:

Of the 3059 women who had a spontaneous vaginal birth, 2831 women had consented to participate in the follow-up questionnaire.

Inclusion criteria were women **opting** for their first vaginal birth from gestational week 37+0 with a singleton pregnancy and a live fetus in the vertex presentation.
Are patients undergoing cesarean section and assisted delivery excluded? I do not see these patients in the flowchart. Please explain.

Author's reply: Women opting for vaginal birth were eligible for the Oneplus trial but only those who were randomized and had a spontaneous vaginal birth were included in the trial. We have now clarified this in the abstract, p 1, line 12:

Inclusion criteria in the Oneplus trial were women opting for their first vaginal birth

Conclusions:
"Women in both groups were generally satisfied with their experiences of the second stage." Please revise. I think better give only percentage.
The conclusion should be revised to align with the objective of the study.

Author's reply: Thank you for this suggestion, we agree that this does not align with the study's objective and have removed the sentence.

STRENGTHS AND LIMITATIONS OF THIS STUDY:
This section must be revised to focus on the study's subject. I suggest retaining only the first sentence and changing the others.

Author's reply: Thank you for your comment. We have reviewed the bullet points and followed the instructions for authors stating that this section should specifically relate to the study's methods.

Introduction: More precision must be given on the role of the second midwife. Why do the authors think it can improve the delivery experience?

Author's reply: To clarify the role of the second midwife, we have added a sentence on p 3, line 19-21 that reads:

The midwives were instructed to use the established prevention models[17,18] at their respective labour ward and for the second midwife to be ready to assist the primary midwife if asked and to support the birthing woman if needed.[16]
We did not believe that the intervention could improve women´s experience of the second stage, rather that it can be negatively affected since previous research shows that it can be difficult to establish new relationships during this stage. We have now described this further on p 3 line 21-25:

Since previous research shows that it can be difficult to establish new relationships during the second stage[1], it can be assumed that the assistance of a second midwife may influence the women´s experiences of the second stage negatively. Therefore, this was predefined as a secondary outcome in the trial.[19]

Methods:
Inclusion criteria were women aged between 18 to 47 who opted for them. Is this the inclusion criteria for results? Why 47 years and not 48?

Author's reply: Based on data from statistics in Sweden on the age of birthing women we considered it unlikely that women over 47 years of age to be eligible for the trial.

I think the authors are confusing this with the study's results.
"Further inclusion criteria were language proficiency in Swedish, English, Arabic, or Farsi, as study information was only available in these languages," and "questionnaire one month after birth, which was available in Swedish and English." This sentence is confusing, as the principal objective of this second analysis of data is based on the questionnaire. Parturient speaking Arabic and Farsi agreed to participate but received questionnaires in non-adapted languages. I think it's better to exclude these patients from the analysis to avoid confusion.

Author's reply: The flowchart gives information that 27 women randomized to two midwives and 18 women randomized to one midwife did not master the Swedish or English language. For clarity, we have added a sentence on p 4, line 14-15:

As the questionnaire was only available in Swedish and English, women who did not master Swedish or English were not included in the current study.

Results: Of the 2831 women who consented to participate in the follow-up, 2233 (78.9%) responded to the questionnaire. Here, patients consented to participate but received non-adapted questionnaires, and I think they should be excluded.

Author's reply: Women who consented to participation in the Oneplus trial had the option to consent or decline participation to receive the follow-up questionnaire one month after birth. All women who consented to receive the questionnaire received the same version, either in Swedish or in English.

Discussion:

"The findings from this study show that women, regardless of randomization, reported overall positive experiences of the second stage of labor." Please revise this sentence as it does not align with the study's objective.

Author's reply: Thank you for pointing this out. We have removed the sentence from the discussion and the conclusion.

"The differences in the incidence of SPT and the length of the second stage of labor between the two groups have been reported earlier, in the results of the Oneplus trial." Please revise this sentence, as a 2-minute difference in the second stage of labor, despite being statistically significant, has limited practical benefit.

Author's reply: We agree that the 2-minute difference has limited relevance. On p 13, line 20-22, we have described that the significant difference in the length of the active second stage in this study appears to be clinically irrelevant for the women.

"A prolonged labor has been associated with negative birth experiences" - where?

Author's reply: This has been shown in a systematic review by Hosseini et al (2020), reference 31.

"However, the significant difference in the length of the active second stage in this study appears to be clinically irrelevant for the women's experiences in this study." Please be cautious in interpreting the results. Do the authors believe that 2 or 3 minutes can change the patient's perception?

Author's reply: Please see our answer above.

Conclusion:

"The findings from this study show that women reported overall positive experiences of the second stage of labor." Please provide a conclusion and remove the phrase "regardless of randomization."

Author's reply: Thank you for pointing this out. We have removed the sentence.

Include "I could handle the situation during the second stage of labor" as it is significant.

Author's reply: Thank you for your comment. We have added two sentences to the conclusion on p 16, line 10-13 that reads:

Women randomised to assistance by two midwives agreed to a lesser extent that they could handle the situation during the second stage of labour. However, the mean difference was small and only significant for one of the study sites.

"as being assisted by two midwives reduces SPT» refers to the results of the same study already published. This information needs to be confirmed by other studies and adapted to the context of application.

Author's reply: Thank you for pointing this out. We have chosen to add two sentences on p 16, line 2-5, to address this limitation:

However, as the trial was conducted in the Swedish setting, the result may not be generalizable to other countries or contexts. To further understand the implication of the intervention on women's experiences, a qualitative study could provide important insights.

Flowchart:
Participants were excluded because "27 did not master the Swedish or English language." These patients must be excluded based on the inclusion criteria of this analysis?

Author's reply: In total 45 women did not master the Swedish or English language and were excluded (27 women randomised to two midwives and 18 women randomised to one midwife). It is correct that they were excluded. However, they were initially included and randomised in the Oneplus trial. To clarify this, the following information has been added on p 4, line 14-15:

As the questionnaire was only available in Swedish and English, hence, women who did not master Swedish or English were not included in the current study.

patients undergoing cesarean section and assisted delivery excluded. Are they retained in the analysis?

Author's reply: Thank you for pointing this out. We have clarified this in the top box in the flowchart which now reads:

3059 women were included in the RCT, gave birth spontaneously, and randomised to standard care (one midwife) or intervention (two midwives during the second stage of labour

RCT: give details of this abbreviation.

Author's reply: Thank you. The abbreviation is added under the flowchart:

RCT=Randomised controlled trial

Reviewer: 5
Dr. Sybil Prince Nelson, Washington and Lee University

Comments to the Author:
To Whom it May Concern,

I have had the opportunity to review the manuscript titled "Impact of collegial midwifery assistance during the second stage of labour on women's experience: A follow-up study from the Oneplus randomised controlled trial" submitted to BMJ Open, and I am pleased to share my evaluation. The article is commendably structured, offering a clear motivation, methodology, results, and conclusions. The subject matter is both interesting and significant, addressing an essential aspect of maternal care.
The study's design is noteworthy, effectively navigating the inherent limitations of non-blinding due to

the intervention method. Despite these constraints, the research provides valuable insights into women's experiences during the second stage of labor.

Author’s reply: Thank you for the time you dedicated to reviewing the manuscript. We are grateful for your valuable feedback.

The manuscript adeptly acknowledges potential biases and the challenges of capturing complex attitudes with single-item measurements.
A key finding of the study, the statistical difference in severe perineal trauma between the intervention and control groups. Although this finding is marginally significant, it remains an interesting aspect of the study. This outcome, suggesting fewer instances of severe perineal trauma in the midwife-assisted group, merits further emphasis within the manuscript. Highlighting this finding could enhance the paper's contribution to the field, especially given the scarcity of other differing outcomes between the compared groups.

Author’s reply: Thank you for the suggestion. We agree that highlighting the difference in SPT needs more emphasis within the manuscript. Therefore, we have elaborated on this findings and added the following text on p 16 line 1-9:

Although we found no differences between women assigned to the intervention and those receiving standard care, a recent study within the Oneplus trial reported that collegial midwifery assistance particularly appreciated by women with fear of birth, those with lower educational attainment, and those who did not have Swedish as their native language.[33] This reinforces the findings from the present study, altogether showing that collegial midwifery assistance is a well-accepted intervention that can be safely implemented into standard care to reduce SPT. However, as the trial was conducted in the Swedish setting, the result may not be generalizable to other countries or contexts. To further understand the implication of the intervention on women’s experiences, a qualitative study could provide important insights.

However, my primary concern lies with the analysis of Likert scale data. The current approach could be refined to strengthen the manuscript's methodological rigor. I recommend a more nuanced evaluation of the Likert data, incorporating strategies such as utilizing median and interquartile range (IQR) for capturing central tendency and variability, employing non-parametric tests like the Mann-Whitney or Wilcoxon for comparisons, and considering ordinal logistic regression for modeling relationships with ordinal responses. Additionally, conducting factor analysis and calculating Cronbach’s alpha could further validate the scale's reliability and dimensionality.
Implementing these suggestions could significantly enhance the paper's analytical depth, offering a more robust understanding of women's experiences during labor. Not all of these methods are necessary, but at least one or two would be beneficial. This, in turn, would bolster the paper's overall strength and its contribution to the existing body of knowledge in maternal healthcare.
Thank you for the opportunity to review this manuscript. I look forward to seeing the revised version and believe that with these adjustments, the paper will make a valuable addition to BMJ Open.

Best regards,

Sybil Prince Nelson

Author’s reply: Thank you for your valuable comment. We have discussed the choice of analyses with our statistician and have now performed Mann Whitney U tests and provided p-values in table 3, p 12. The results section and statistical analyses have been changed accordingly. However, due to the randomised design and no significant differences between the groups regarding background characteristics (Table 1), we cannot find the added value of adjusting in an ordinal logistic regression

model. We agree that it would be of value in future research to do psychometric testing of the items used in this questionnaire which we have addressed in the strengths and limitation section, p 15, line 12-16.

VERSION 2 – REVIEW

**REVIEWER**	Schwind, Bettina<br>Kalaidos University of Applied Sciences, Careum School of Health
**REVIEW RETURNED**	19-May-2024

**GENERAL COMMENTS**	Reviewer: 1<br>Dr. Bettina Schwind, Kalaidos University of Applied Sciences, Swiss Tropical and Public Health Institute, University of Zurich<br><br>Comments to the Author:<br>It was a pleasure to review the manuscript entitled "Impact of collecgial midwifery assistance during second stage of labour on women's experiences: A follow-up study from the Oneplus randomized controlled trial". The overall aim of the study was to compare the experience of the second stage of labour between women assigned to collegial midwifery and standard care. The study is important because it reports women's experiences of the intervention, which is key to ensuring high quality care and preventing trauma. It also adds well to previously published data from the Oneplus trial.<br><br>Author's reply: Thank you. We are grateful for your time in reviewing the article and for your positive feedback.<br><br>Overall, the manuscript is clearly structured and reads well, but I have some suggestions for improvement. In my view, a key aspect is to make more explicit which type of women's experiences and/or which dimensions (control? vulnerability? pain? other?) were assessed for which reasons. This would improve the overall clarity of the manuscript's message and may need to be integrated throughout (methods, results and discussion - see other comments below).<br><br>Author's reply: Thank you for your comment. The items are based on factors that previously has been identified as being of significance for the birth experience and/or the experience of the second stage of labour, such as the woman's feelings of safety and of being in control. This was done by carrying out a review of the literature and by discussions within the research group with contemporary clinical expertise, which is described on p. 5, line 5-10.<br><br>Reply from reviewer:

	Thanks for your valuable clarification. The process (review and research group discussion) is well described. It would be even clearer if you could describe in detail which main themes/items/dimensions you identified through the literature review and which were added by the research group. This would greatly enhance the reader's understanding of the questionnaire items.<br><br>TITEL<br>I suggest changing the title of the study slightly to make it more precise: "Impact of collegial midwifery support on women's experiences of the second stage of labour".<br><br>Author's reply: Thank you for your suggestion. The term assistance has been used in several articles1-5 regarding the intervention, including the study protocol, why we have decided to keep the term assistance in the title and throughout the manuscript.<br><br>Edqvist M, Dahlen HG, Häggsgård C, Tern H, Ängeby K, Tegerstedt G, Teleman P, Ajne G, Rubertsson C. One Plus One Equals Two-will that do? A trial protocol for a Swedish multicentre randomised controlled trial to evaluate a clinical practice to reduce severe perineal trauma {1}. Trials. 2020 Nov 23;21(1):945. doi: 10.1186/s13063-020-04837-7. Erratum in: Trials. 2020 Dec 2;21(1):993. PMID: 33225972; PMCID: PMC7682019.<br><br>Edqvist M, Dahlen HG, Häggsgård C, Tern H, Ängeby K, Teleman P, Ajne G, Rubertsson C. The effect of two midwives during the second stage of labour to reduce severe perineal trauma (Oneplus): a multicentre, randomised controlled trial in Sweden. Lancet. 2022 Mar 26;399(10331):1242-1253. doi: 10.1016/S0140-6736(22)00188-X. Epub 2022 Mar 15. PMID: 35303474.<br><br>Tern H, Edqvist M, Ekelin M, Dahlen HG, Rubertsson C. Swedish midwives' experiences of collegial midwifery assistance during the second stage of labour: A qualitative study. Women Birth. 2023 Feb;36(1):72-79. doi: 10.1016/j.wombi.2022.03.003. Epub 2022 Mar 17. PMID: 35307300.<br><br>Tern H, Edqvist M, Ekelin M, Dahlen HG, Rubertsson C. Primary midwives' experiences of collegial midwifery assistance during the active second stage of labor: Data from the Oneplus trial. Birth. 2023 Dec;50(4):868-876. doi: 10.1111/birt.12739. Epub 2023 Jul 4. PMID: 37401365.

Tern H, Rubertsson C, Ekelin M, Dahlen HG, Häggsgård C, Edqvist M. Women's experiences of being assisted by two midwives during the active second stage of labour: Secondary outcomes from the Oneplus trial. Sex Reprod Healthc. 2024 Mar;39:100926. doi: 10.1016/j.srhc.2023.100926. Epub 2023 Nov 24. PMID: 38041929.

Reply from reviewer:

Thanks for your valuable clarification.

ABSTRACT

• I suggest a slight revision of the abstract for clarity - delete the second part "during the second stage of labour" and move the "Oneplus trial" to the methods (p.2 lines 13-17).

Author’s reply: Thank you, we have changed this in the abstract.

Reply from reviewer:

Thanks for your changes.

• Please change the methods accordingly. I would suggest highlighting in the results section that the "handling of the situation" was largely due to a setting.

Author’s reply: Thank you for your comment. We have changed the Design section in the abstract, p1, line 9-10, which now reeds:

Design Analysis of a secondary outcome within the Swedish Oneplus multicenter randomised trial.

We have also added the following text in the results section in the abstract p 1, line 29-30:

Conducted sub-group analyses revealed that this result originated from one of the study sites.

• I would also suggest deleting the first sentence of the conclusion (p.3, line 4), as it is not clear from the manuscript whether the measured dimensions necessarily allow the assumption of general satisfaction in both arms.

	Author’s reply: Thank you for pointing this out. We agree and have deleted this sentence.<br><br>Reply from reviewer:<br><br>Thanks for your changes.<br><br>Strengths and limitations:<br>• Please, specify why the RCT as design is a strength (no reasons are given, also in the discussion section (p.14/line 45).<br><br>Author’s reply: In the strength and limitation section after the abstract, the first bulletpoint, p 2, line 8-9, now reads:<br><br>The randomised design is considered the most rigorous and robust design for determining cause and effect.<br><br>We have also specified this in the discussion section on p 15, line 3-4<br><br>Strengths of this study include the high response rate to the questionnaire and the randomised design36 which is considered the most rigorous and robust design for determining a cause and effect.37<br><br>Reply from reviewer:<br><br>Thanks for your changes; please specific that an RCT is most rigorous to determining cause and effect between an intervention and an outcome.<br><br>Also, from the methods part, the patient involvement throughout the study (design and implemention) is not made fully clear (see comments in methods).<br><br>As a limitation; I would suggest to include that there was no masking (midwifes and patients) done/possible which might have introduced bias (especially on women’s experiences).

	Author's reply: Thank you for this suggestion. We have added the following bulletpoint, p 2, line 12-13:<br><br>It was not possible to blind the participants or midwives due to the nature of the intervention which may have influenced women's responses.<br><br>Reply from reviewer:<br><br>Thanks for your changes.<br><br>INTRODUCTION<br>• p. 3 line 40/41 "During the second stage of labour…" might be deleted.<br><br>Author's reply: Thank you for your comment, the sentence is changed according to your suggestion.<br><br>Reply from reviewer:<br><br>Thanks for your changes.<br><br>• p.5 line 11: I suggest referring directly to the Oneplus study.<br><br>Author's reply: We have referred to the Oneplus trial on p. 4, line 12, and changed the sentence:<br><br>Further inclusion criteria in the Oneplus trial were language proficiency in Swedish, English, Arabic or Farsi as study information was only available in these languages.<br><br>Reply from reviewer:<br><br>Thanks for your changes.<br><br>METHODS<br><br>Study design<br>• P.5 lines 41-47: "This study...by a midwife.", repetition of the aims of the present study and the context of the Oneplus trial, so please either delete or move aspects you consider important to the introduction.<br><br>Author's reply: Thank you for observing this. We have deleted the two sentences.

	• Please include a sentence stating that no masking of midwives/women was possible.<br><br>Reply from reviewer:<br><br>Thanks for your changes.<br><br>Author’s reply: Thank you for pointing this out. We have now added a sentence about blinding on p 15, line 10-11, that reads:<br><br>Firstly, due to the nature of the intervention, it was not possible to blind the participating women or the midwives.<br><br>Reply from reviewer:<br><br>Thanks for your changes.<br><br>• P.5, line 7: Is it true that the information material was available in English, Swedish, Arabic and Farsi, but the questionnaire was only available in English and Swedish? If so, please give reasons for this.<br><br>Author’s reply: It is correct that information was available in the four languages but that the questionnaire only was available in English and Swedish. Our intention was to translate the questionnaire to all four languages, however, for pragmatic reasons and limited funding this was not possible. We have added a sentence to clarify this, p 4, line 14-15:<br><br>As the questionnaire was only available in Swedish and English, hence, women who did not master Swedish or English were not included in the current study.<br><br>Reply from reviewer:<br><br>Thanks for your changes.<br><br>Questionnaire to women one month after birth<br>• p.6. line 34: I suggest changing the subtitle to “Questionnaire development”

	Author’s reply: Thank you, we have changed the subtitle according to your suggestion.<br><br>Reply from reviewer:<br><br>Thanks for your changes.<br><br>• p.6. line 39/38 and line 44/45: the questionnaire was developed to assess women´s experiences of the second stage of labour and collegial midwifery assistance (intervention), perineal pain, initiation of breastfeeding, and postnatal depression. The wording is a little confusing and needs some clarification, as the study analysed the experiences of the second stage and these are shown in box 1. Please remove the 'experience of collegial midwifery support' (underlined above) as this was probably not part of the questionnaire developed but was intended to be assessed. For the sake of clarity, I will assume that perineal pain refers to the post-partum period. A number of items in box 1 ask about the experience of pain. Therefoe, I suggest that the sentence be slightly revised to help the reader to follow more quickly: e.g. "Experiences of the second stage of labour (intervention experiences) and perineal pain...(postpartum experiences)".<br><br>Author’s reply: Thank you for your suggestion. We have changed the sentences on p 4, line 32 – p 5, line 2, for clarity:<br><br>A study specific questionnaire was developed to assess secondary outcomes in the trial; experiences of the second stage of labour and collegial midwifery assistance. Furthermore, questions regarding sociodemographic background and history of physical and mental health. For the purpose of the present study, the items regarding experiences of the second stage were analysed (Box 1).<br><br>Reply from reviewer:<br><br>Thanks for your changes.<br><br>• p.6. line 55 to p.7 line 16, including box 1: Please describe in more detail how the questionnaire was developed: The aim of the study was to assess women's experiences of the second stage of labour. However, the results of the literature review (key dimensions of women's experiences) remain unclear, and which dimensions have been identified or added by experts as relevant to the second stage? And how did these processes contribute to the items in Box 1? Specifically, which item contributes to which dimension/attribute of women's experiences that you have identified as relevant for the second stage? Which I assume from reading the results/conclusion are 'control', 'vulnerability' and 'pain', but also others. Please explain the development of the

	questionnaire in the text, but also add the overall dimensions in box 1, as this would help the reader to better understand your results.<br><br>Author's reply: Thank you for your comment. The items are based on factors that previously has been identified as being of significance for the birth experience and/or the experience of the second stage of labour, such as the womans' feelings of safety and of being in control. This was done by carrying out a review of the literature and by discussions within the research group with contemporary clinical expertise, which is described on p. 5, line 5-10. We agree that it would be of value to add dimensions. However, in this study psychometric testing was not performed to identify dimensions and suggest this to be carried out in future research.<br><br>Reply from reviewer:<br><br>See comment above, thank you.<br><br>• Another aspect that needs to be clarified in relation to the development of the questionnaire is the 'involvement' of women, as compared to the 'patient and public involvement' section (p.8, line 59 - p.8, line 4): Here you mention that women were involved in the development of the questionnaire, but this is not mentioned and explained in the methods section. This needs to be included in the development of the questionnaire and/or excluded from the PPI section, as well as the strengths/limitations. Could you also add some information about the women? For example, did they receive collegial midwifery support? How many children were born? And how were they recruited/sampled?<br><br>Author's reply: In the methods section, p 5, line 10-13, we have described that ten women who had recently given birth reviewed the questionnaire using a think aloud process with cognitive interviewing. We have added a sentence on p 5, line 13-16, to include more information about the women:<br><br>The women were recruited at the postnatal ward and postnatally in primary care. Individual interviews were carried out 4-10 weeks after birth. Of the ten women recruited, nine women were primiparous, and one woman had given birth after a previous caesarean section.<br><br>Reply from reviewer:<br><br>Thanks for your changes.

	• Box 1: Please add the dimensions of experience and allocate the corresponding items.<br><br>Author’s reply: Thank you for your suggestion. However, in this study we have used single items. As described under strengths and limitations, using a psychometrically tested scale could have strengthened the result but this was not possible since no scale report specifically on experiences of the second stage of labour.<br><br>Reply from reviewer:<br><br>Thanks for your changes.<br><br>• This section does not report on a pre-test of the questionnaire. If this is the case, you may want to add this to the limitations; and/or give reasons why this was not necessary?<br><br>Author’s reply: Thank you for addressing this. We have added the following text under the strength and limitations section, p 15, line 12-14:<br><br>Secondly, although the items in the questionnaire were tested for face validity, no further measures were undertaken to validate the items.<br><br>Reply from reviewer:<br><br>Thanks for your changes.<br><br>Data collection<br>• It is not entirely clear who received how many reminders and how (postal / online / SMS?) There seem to be differences between the English and Swedish language groups; you can also specify when you send reminders (one week after...).<br><br>Author’s reply: On p 6, line 9-12 we have now clarified how many reminders were sent, when they were sent and how:<br><br>To increase participation, four reminders were sent out to non-responders with one-week intervals between each. The first two reminders were sent as text messages, the third reminder was sent as a postal survey and the fourth reminder was sent as a text message.

	Reply from reviewer:<br><br>Thanks for your changes.<br><br>Patient and public involvement<br>• See comment in methods section: If the ten women were not involved in the design of the questionnaire, but only in the validation of the questionnaire, this cannot be considered PPI. Similarly, dissemination of knowledge per se is not considered PPI unless the patients/public are actively involved in the dissemination process and have a say in writing, dissemination and deciding what is relevant to other women. So please consider clarifying or revising.<br><br>Author's reply: Thank you for your comment. We have deleted "and by including ten women in developing and validating the follow-up questionnaire" in the PPI section. Furthermore, we have now stated that women will not be included in the dissemination process on p. 7, line 27-28:<br><br>However, they will not participate in disseminating the results to the public.<br><br>Reply from reviewer:<br><br>Thanks for your changes.<br><br>RESULTS<br><br>Background and labour birth characteristics<br>• P9 line 47: You compare the current results to those of the Oneplus trial, please provide the reference here.<br><br>Author's reply: Thank you for observing this. The reference has now been added. P 8, line 22.<br><br>Reply from reviewer:<br><br>Thanks for your changes.<br><br>Women's experiences of the second stage of labour<br>• P. 11 line 54/55: You report the results of the questionnaire, which you may (slightly) revise after revising the methods/box 1, including dimensions, e.g. also be precise - e.g. you use different wording throughout the manuscript (e.g. feelings, memories, experiences) and also, you do not mention vulnerability here, but in the summary of the discussion you do.

	Author's reply: Thank you for observing this. We have added "vulnerability" to the sentence on p 10, line 10:<br><br>There were no statistically significant differences in women's experiences concerning feelings of control, vulnerability, pain, and memories during the second stage of labour between the two groups.<br><br>The wording refers to the items which include feelings (i.e. I felt strong during the second stage of labor), memories (i.e. I have positive memories from the second stage of labor) and experiences (i.e. How did you experience the length of the second stage of labor?)<br><br>Reply from reviewer:<br><br>Thanks for your changes.<br><br>DISCUSSION<br>• P. 14 line 12-21 (summary): I would suggest not summarising the results as positive experiences, as this cannot be fully assumed from the results. I suggest staying with "The results of the study show no significant differences...". I also suggest adding that the differences found in handling the situation were due to one side of the study (see commentary abstract).<br><br>Author's reply: Thank you for your suggestion. The sentence on p. 13, line 3-5 has been changed and now reads:<br><br>The findings from this study show that there were no statistically significant differences between the groups regarding women's experiences of pain, feelings of vulnerability or being in control and experiences of the length second stage of labour.<br><br>Furthermore, we have added the following wording to the sentence on p. 13, line 8-9.<br><br>However, women randomised to assistance by two midwives agreed to a lesser extent that they could handle the situation during the second stage of labour. Conducted sub-group analyses revealed that this result originated from one of the study sites.<br><br>Reply from reviewer:

	Thanks for your changes.<br><br>• P 14. Line 23-30: Please be specific, add and elaborate, as the comparison with the existing RCTs is a bit short, e.g. the RCTs had very different interventions? What kind of women's experiences were assessed? Can you really compare it with yours? There are also RCTs that have had a positive impact on women's experiences (e.g. https://doi.org/10.1007/s00404-023-07115-4).<br><br>Author’s reply: Thank you for your comment. We have added two sentences on p 13, r 13-18 to further elaborate the comparison with the RCTs.<br><br>The questionnaires used to measure birth experience in these trials include similar items as in the present study. In those trials The intervention under investigation had little or no impact on women’s birth experiences in these trials. This might be explained by the fact that other factors such as support from caregivers and feelings of being safe and in control and participation in decision-making may be more important for the birth experience.27,28<br><br>Reply from reviewer:<br><br>Thanks for your changes.<br><br>• P14, line 33-57: Another reason may be that the second stage of labour is relatively short compared to the total length of labour; continuity of care and the relationship with the midwife, but also communication (MIMA model) are also key to women's experience of labour. Such aspects could be further reflected here, also in that the second midwife may not have been perceived as an 'interruption' (understood my needs) and thus changed the overall experience.<br><br>Author’s reply: Thank you for this suggestion. On p 14, line 18-12, we have discussed the significance of effective communication for the birth experience and referred to a study by Tern et al (2024) showing that few women felt disrupted by the second midwife.<br><br>However, a recent published study within the Oneplus trial showed that among women actually receiving the intervention, only 6,7% of the women were negative towards being assisted by an additional midwife during the second stage.33

	Reply from reviewer:<br><br>Thanks for your changes.<br><br>• Overall, I suggest adding a section on implications for clinical practice and research/unanswered questions, which has not been reflected in the discussion so far.<br><br>Author's reply: Thank you for pointing this out. On p 16, line 1-7, we have reflected on the implications for clinical practice:<br><br>Although we found no differences between women assigned to the intervention and those receiving standard care, a recent study within the Oneplus trial reported that collegial midwifery assistance particularly appreciated by women with fear of birth, those with lower educational attainment, and those who did not have Swedish as their native language.33 This reinforces the findings from the present study, altogether showing that collegial midwifery assistance is a well-accepted intervention that can be safely implemented into standard care to reduce SPT.<br><br>Reply from reviewer:<br><br>Thanks for pointing this out.<br><br>Strengths and limitations<br><br>• P15 line 44: Why is the RCT considered a strength, please include a short explanation.<br><br>Author's reply: We have added an explanation on p 15, line 2-4, that reads:<br><br>Strengths of this study include the high response rate to the questionnaire and the randomised design36 which is considered the most rigorous and robust design for determining a cause and effect.37<br><br>Reply from reviewer:<br><br>Thanks for your changes, please see comment above on RCT.<br><br>• P15 line 50: The involvement of women in the design of the questionnaire is not described in the methods. Please, revise accordingly.

	Author’s reply: The involvement of women in the design of the questionnaire is described in the methods section, p 5, line 10-16.<br><br>To test the validity and the relevance of the items in the questionnaire, ten women who had recently given birth were invited to review the questionnaire using a think aloud process with cognitive interviewing.22,23 The women were recruited at the postnatal ward and postnatally in primary care. Individual interviews were carried out 4-10 weeks after birth. Of the ten women recruited, nine women were primiparous, and one woman had given birth after a previous caesarean section.<br><br>Reply from reviewer:<br><br>Thanks for your changes.<br><br>• P15 line 59: Midwives were also aware, right? Please add to the bias.<br><br>Author’s reply: Thank you for observing this. The sentence on p. 15, line 10-11. is changed and reads:<br><br>Firstly, due to the nature of the intervention, it was not possible to blind the participating women or the midwives.<br><br>Reply from reviewer:<br><br>Thanks for your changes.<br><br>• Please add to the limitations that the questionnaire was not pre-tested.<br><br>Author’s reply: Thank you for your suggestion. We have now added to the limitations that no further measures were undertaken to validate the items. P 15, line 12-16.<br><br>Secondly, although the items in the questionnaire were tested for face validity, no further measures were undertaken to validate the items.

	Reply from reviewer:<br><br>Thanks for your changes.<br><br>• P16 line 16-18: In order to better understand women's experiences, a follow-up qualitative study could have been added to better understand the wider implications in more depth, as in the dimensions of 'coping with the situation', which you may want to add as a limitation, also in terms of what collegial midwife means or changes in women's experiences.<br><br>Author's reply: We agree that a qualitative study can add a deeper understanding and have added this on p. 16, line 8-9.<br><br>To further understand the implication of the intervention on women's experiences, a qualitative study could provide important insights.<br><br>Reply from reviewer:<br><br>Thanks for your changes.<br><br>CONCLUSION<br>As mentioned above, I would recommend not to report 'positive' experiences, but to stay with the findings as in differences/similarities found, and to report the respective dimensions, and to conclude from this the implications for practice here (see discussion section).<br><br>Author's reply: Thank you. The conclusion has been changed according to your suggestion.<br><br>Reply from reviewer:<br><br>Thanks for your changes.<br><br>Reviewer: 2<br>Dr. Nabila H. Purno, United Nations Population Fund<br><br>Comments to the Author:<br>This is a very clear and well written article- congratulations to the author for researching on this topic and preparing the manuscript.<br><br>Author's reply: Thank you. We are grateful for your time in reviewing the article and for your positive feedback.

	Some minor comments: in the introduction, to describe usual causes of SPT, a bit on how women's experience in the second stage of labor can contribute to SPT.<br><br>Author’s reply: Thank you for your suggestion. We have added causes to SPT on p 3, line 1-2.<br><br>Risk factors for SPT include nulliparity, first vaginal birth after caesarean section, instrumental birth, increased fetal birthweight, increased maternal age and Asian ethnicity.10,11<br><br>The authors can also describe a little bit about collegial midwifery assistance and what is the theoretical basis for them to assume that two midwives should improve or impact labour experiences and SPT.<br><br>Author’s reply: Thank you. We have added the following sentence regarding collegial midwifery assistance on p 3, line 19-21:<br><br>The midwives were instructed to use the established prevention models17,18 at their respective labour ward and for the second midwife to be ready to assist the primary midwife if asked and to support the birthing woman if needed.16<br><br>However, we did not assume that the intervention should improve the experience of the second stage of labour, rather that it can be negatively affected. We have clarified this on p 3, line 21-24:<br><br>Since previous research shows that it can be difficult to establish new relationships during the second stage1, it can be assumed that the assistance of a second midwife may influence the women´s experiences of the second stage negatively.<br><br>In the results section, it would have been good to have some reference to neonatal birth outcomes - since that may also have introduced recall bias.<br><br>Author’s reply: There were no differences in the neonatal outcomes (Table 2). We have added a sentence in the result on p 8, line 27-28:

	The incidence of Apgar score below 7 was low with no statistical difference between the groups.<br><br>In the discussion section,<br>Other than these, the paper is very clear and articulate.<br><br>Reviewer: 3<br>Dr. Marie Oscarsson, Linnaeus University<br>Comments to the Author:<br>Congratulations for your large work – the subject of the study is of interest especially in midwifery practice. I have only one suggestion-Please, do not repeat the results from tables. This section would be more fluent to read.<br><br>Author's reply: Thank you for your comment and your positive feedback. We have deleted results from the tables that were duplicated in the text and hope it is more fluent to read now.<br><br>Reviewer: 4<br>Dr. Najeh Hcini, West French Guiana Hospital Center<br>Comments to the Author:<br>see attached file<br><br>This is an interesting study in obstetrics, and an experience to be shared with perinatal care providers.<br><br>Author's reply: We are grateful for your feedback and for your time in reviewing the article.<br><br>This paper merits revision before publication, especially as regards the methodology and the inclusion/exclusion process. Flowchart should be revised.<br><br>Inclusion: 'opting for their first vaginal birth'; exclusion: 'planned cesarean section.' The authors don't mention patients undergoing emergency cesarean section and assisted vaginal delivery.<br><br>Author's reply: Thank you for your comment. We have referred to the Oneplus trial on p 4, line 10, for full details.<br><br>Abstract<br><br>Participants: Please provide information on the inclusion or exclusion criteria for the induction of labor. If a cesarean is performed, please provide more precision. Of the 3059 women

	who gave birth spontaneously, 2831 women consented to participate in the follow-up questionnaire. Please clarify the term 'spontaneously.'<br><br>Author's reply: Thank you for your suggestion. We have clarified the term spontaneously on p 1, line 16-17:<br><br>Of the 3059 women who had a spontaneous vaginal birth, 2831 women had consented to participate in the follow-up questionnaire.<br><br>Inclusion criteria were women opting for their first vaginal birth from gestational week 37+0 with a singleton pregnancy and a live fetus in the vertex presentation.<br><br>Are patients undergoing cesarean section and assisted delivery excluded? I do not see these patients in the flowchart. Please explain.<br><br>Author's reply: Women opting for vaginal birth were eligible for the Oneplus trial but only those who were randomized and had a spontaneous vaginal birth were included in the trial. We have now clarified this in the abstract, p 1, line 12:<br><br>Inclusion criteria in the Oneplus trial were women opting for their first vaginal birth<br><br>Conclusions:<br><br>"Women in both groups were generally satisfied with their experiences of the second stage." Please revise. I think better give only percentage.<br><br>The conclusion should be revised to align with the objective of the study.<br><br>Author's reply: Thank you for this suggestion, we agree that this does not align with the study's objective and have removed the sentence.<br><br>STRENGTHS AND LIMITATIONS OF THIS STUDY:<br><br>This section must be revised to focus on the study's subject. I suggest retaining only the first sentence and changing the others.

	Author's reply: Thank you for your comment. We have reviewed the bullet points and followed the instructions for authors stating that this section should specifically relate to the study's methods.<br><br>Introduction: More precision must be given on the role of the second midwife. Why do the authors think it can improve the delivery experience?<br><br>Author's reply: To clarify the role of the second midwife, we have added a sentence on p 3, line 19-21 that reads:<br><br>The midwives were instructed to use the established prevention models17,18 at their respective labour ward and for the second midwife to be ready to assist the primary midwife if asked and to support the birthing woman if needed.16<br><br>We did not believe that the intervention could improve women´s experience of the second stage, rather that it can be negatively affected since previous research shows that it can be difficult to establish new relationships during this stage. We have now described this further on p 3 line 21-25:<br><br>Since previous research shows that it can be difficult to establish new relationships during the second stage1, it can be assumed that the assistance of a second midwife may influence the women´s experiences of the second stage negatively. Therefore, this was predefined as a secondary outcome in the trial.19<br><br>Methods:<br><br>Inclusion criteria were women aged between 18 to 47 who opted for them. Is this the inclusion criteria for results? Why 47 years and not 48?<br><br>Author's reply: Based on data from statistics in Sweden on the age of birthing women we considered it unlikely that women over 47 years of age to be eligible for the trial.<br><br>I think the authors are confusing this with the study's results.<br><br>"Further inclusion criteria were language proficiency in Swedish, English, Arabic, or Farsi, as study information was only available in these languages," and "questionnaire one month after birth, which was available in Swedish and English." This sentence is confusing,

	as the principal objective of this second analysis of data is based on the questionnaire. Parturient speaking Arabic and Farsi agreed to participate but received questionnaires in non-adapted languages. I think it's better to exclude these patients from the analysis to avoid confusion.<br><br>Author’s reply: The flowchart gives information that 27 women randomized to two midwives and 18 women randomized to one midwife did not master the Swedish or English language. For clarity, we have added a sentence on p 4, line 14-15:<br><br>As the questionnaire was only available in Swedish and English, women who did not master Swedish or English were not included in the current study.<br><br>Results: Of the 2831 women who consented to participate in the follow-up, 2233 (78.9%) responded to the questionnaire. Here, patients consented to participate but received non-adapted questionnaires, and I think they should be excluded.<br><br>Author’s reply: Women who consented to participation in the Oneplus trial had the option to consent or decline participation to receive the follow-up questionnaire one month after birth. All women who consented to receive the questionnaire received the same version, either in Swedish or in English.<br><br>Discussion:<br><br>"The findings from this study show that women, regardless of randomization, reported overall positive experiences of the second stage of labor." Please revise this sentence as it does not align with the study's objective.<br><br>Author’s reply: Thank you for pointing this out. We have removed the sentence from the discussion and the conclusion.<br><br>"The differences in the incidence of SPT and the length of the second stage of labor between the two groups have been reported earlier, in the results of the Oneplus trial." Please revise this sentence, as a 2-minute difference in the second stage of labor, despite being statistically significant, has limited practical benefit.

	Author’s reply: We agree that the 2-minute difference has limited relevance. On p 13, line 20-22, we have described that the significant difference in the length of the active second stage in this study appears to be clinically irrelevant for the women.<br><br>"A prolonged labor has been associated with negative birth experiences" - where?<br><br>Author’s reply: This has been shown in a systematic review by Hosseini et al (2020), reference 31.<br><br>"However, the significant difference in the length of the active second stage in this study appears to be clinically irrelevant for the women’s experiences in this study." Please be cautious in interpreting the results. Do the authors believe that 2 or 3 minutes can change the patient's perception?<br><br>Author’s reply: Please see our answer above.<br><br>Conclusion:<br><br>"The findings from this study show that women reported overall positive experiences of the second stage of labor." Please provide a conclusion and remove the phrase "regardless of randomization."<br><br>Author’s reply: Thank you for pointing this out. We have removed the sentence.<br><br>Include "I could handle the situation during the second stage of labor" as it is significant.<br><br>Author’s reply: Thank you for your comment. We have added two sentences to the conclusion on p 16, line 10-13 that reads:<br><br>Women randomised to assistance by two midwives agreed to a lesser extent that they could handle the situation during the second stage of labour. However, the mean difference was small and only significant for one of the study sites.

	"as being assisted by two midwives reduces SPT» refers to the results of the same study already published. This information needs to be confirmed by other studies and adapted to the context of application.<br><br>Author’s reply: Thank you for pointing this out. We have chosen to add two sentences on p 16, line 2-5, to address this limitation:<br><br>However, as the trial was conducted in the Swedish setting, the result may not be generalizable to other countries or contexts. To further understand the implication of the intervention on women’s experiences, a qualitative study could provide important insights.<br><br>Flowchart:<br><br>Participants were excluded because "27 did not master the Swedish or English language." These patients must be excluded based on the inclusion criteria of this analysis?<br><br>Author’s reply: In total 45 women did not master the Swedish or English language and were excluded (27 women randomised to two midwives and 18 women randomised to one midwife). It is correct that they were excluded. However, they were initially included and randomised in the Oneplus trial. To clarify this, the following information has been added on p 4, line 14-15:<br><br>As the questionnaire was only available in Swedish and English, hence, women who did not master Swedish or English were not included in the current study.<br><br>patients undergoing cesarean section and assisted delivery excluded. Are they retained in the analysis?<br><br>Author’s reply: Thank you for pointing this out. We have clarified this in the top box in the flowchart which now reads:<br><br>3059 women were included in the RCT, gave birth spontaneously, and randomised to standard care (one midwife) or intervention (two midwives during the second stage of labour<br><br>RCT: give details of this abbreviation.

	Author's reply: Thank you. The abbreviation is added under the flowchart:<br><br>RCT=Randomised controlled trial<br><br>Reviewer: 5<br>Dr. Sybil Prince Nelson, Washington and Lee University<br><br>Comments to the Author:<br>To Whom it May Concern,<br><br>I have had the opportunity to review the manuscript titled "Impact of collegial midwifery assistance during the second stage of labour on women's experience: A follow-up study from the Oneplus randomised controlled trial" submitted to BMJ Open, and I am pleased to share my evaluation. The article is commendably structured, offering a clear motivation, methodology, results, and conclusions. The subject matter is both interesting and significant, addressing an essential aspect of maternal care.<br>The study's design is noteworthy, effectively navigating the inherent limitations of non-blinding due to the intervention method. Despite these constraints, the research provides valuable insights into women's experiences during the second stage of labor.<br><br>Author's reply: Thank you for the time you dedicated to reviewing the manuscript. We are grateful for your valuable feedback.<br><br>The manuscript adeptly acknowledges potential biases and the challenges of capturing complex attitudes with single-item measurements.<br>A key finding of the study, the statistical difference in severe perineal trauma between the intervention and control groups. Although this finding is marginally significant, it remains an interesting aspect of the study. This outcome, suggesting fewer instances of severe perineal trauma in the midwife-assisted group, merits further emphasis within the manuscript. Highlighting this finding could enhance the paper's contribution to the field, especially given the scarcity of other differing outcomes between the compared groups.<br><br>Author's reply: Thank you for the suggestion. We agree that highlighting the difference in SPT needs more emphasis within the manuscript. Therefore, we have elaborated on this findings and added the following text on p 16 line 1-9:

	Although we found no differences between women assigned to the intervention and those receiving standard care, a recent study within the Oneplus trial reported that collegial midwifery assistance particularly appreciated by women with fear of birth, those with lower educational attainment, and those who did not have Swedish as their native language.33 This reinforces the findings from the present study, altogether showing that collegial midwifery assistance is a well-accepted intervention that can be safely implemented into standard care to reduce SPT. However, as the trial was conducted in the Swedish setting, the result may not be generalizable to other countries or contexts. To further understand the implication of the intervention on women's experiences, a qualitative study could provide important insights.<br><br>However, my primary concern lies with the analysis of Likert scale data. The current approach could be refined to strengthen the manuscript's methodological rigor. I recommend a more nuanced evaluation of the Likert data, incorporating strategies such as utilizing median and interquartile range (IQR) for capturing central tendency and variability, employing non-parametric tests like the Mann-Whitney or Wilcoxon for comparisons, and considering ordinal logistic regression for modeling relationships with ordinal responses. Additionally, conducting factor analysis and calculating Cronbach's alpha could further validate the scale's reliability and dimensionality.<br>Implementing these suggestions could significantly enhance the paper's analytical depth, offering a more robust understanding of women's experiences during labor. Not all of these methods are necessary, but at least one or two would be beneficial. This, in turn, would bolster the paper's overall strength and its contribution to the existing body of knowledge in maternal healthcare.<br>Thank you for the opportunity to review this manuscript. I look forward to seeing the revised version and believe that with these adjustments, the paper will make a valuable addition to BMJ Open.<br><br>Best regards,<br><br>Sybil Prince Nelson<br><br>Author's reply: Thank you for your valuable comment. We have discussed the choice of analyses with our statistician and have now performed Mann Whitney U tests and provided p-values in table 3, p 12. The results section and statistical analyses have been changed accordingly. However, due to the randomised design and no significant differences between the groups regarding background characteristics (Table 1), we cannot find the added value of adjusting in an ordinal logistic regression model. We agree that it would be of value in future research to do psychometric testing of the items used in this questionnaire which we have addressed in the strengths and limitation section, p 15, line 12-16.

**REVIEWER**	Purno, Nabila H.<br>United Nations Population Fund
**REVIEW RETURNED**	22-May-2024

**GENERAL COMMENTS**	Thank you for trying your best to sufficiently and diligently address the comments of the reviewers.
